# Vibration Environment Reconstruction for FMCW Radar Using a Radar-Performance-Oriented Spectral Operator

**DOI:** 10.3390/s26216697

**Published:** 2026-10-23

**Authors:** Yuzheng Zhang, Yijie Chen, Chen Ai, Mingming Dong

**Affiliations:** 1 School of Mechatronical Engineering, Beijing Institute of Technology, Beijing 100081, China; 3220220371@bit.edu.cn (Y.Z.); chenyijie@bit.edu.cn (Y.C.); 2 China North Vehicle Research Institute, Beijing 100072, China; 3 School of Mechanical Engineering, Beijing Institute of Technology, Beijing 100081, China; 1120202170@bit.edu.cn

**Keywords:** FMCW radar, tracked vehicle, vibration environment reconstruction, radar performance evaluation, spectral constraint, vibration-induced degradation

## Abstract

Tracked vehicles operating under off-road conditions subject onboard frequency-modulated continuous-wave (FMCW) radars to broadband and impulsive vibration, making vibration-environment reconstruction important for repeatable laboratory evaluation of vibration-induced sensing degradation. Conventional reconstruction methods mainly target vibration-signal characteristics, such as power spectral density (PSD), root mean square (RMS), and higher-order statistics, without explicitly accounting for the frequency-selective influence of vibration components through the radar processing chain. This study proposes a radar-performance-oriented spectral operator for FMCW radar velocity measurement and incorporates it, together with vibration PSD and higher-order statistical features, into the reconstruction process. Numerical simulations assess reconstruction performance, and shaker tests assess laboratory implementation and consistency with a model-based radar reference. Compared with reconstruction based on PSD and higher-order statistics, the proposed method reduces the mean relative $L_{2}$ error of the sensitive-band effective-energy vector from 38.07% to 16.95%. In simulation, the reconstructed main-peak broadening and spurious-peak center position are 1.26m/s and 0.68m/s, corresponding to relative errors of 4.13% and 1.45%, respectively, with respect to the simulated original-vibration response. In shaker tests, the corresponding measured values are 1.133m/s and 0.637m/s, with relative deviations of 6.36% and 7.68%, respectively, from the simulation reference obtained using the original measured vibration input. The results support improved agreement with the model-based reference in the evaluated cases while maintaining comparable vibration statistical characteristics. Accuracy relative to actual field radar degradation remains unverified because an independent field-measured radar baseline is unavailable.

## 1. Introduction

Frequency-modulated continuous-wave (FMCW) millimeter-wave radar offers advantages such as strong environmental adaptability, non-contact measurement, and direct acquisition of radial velocity, and has been widely applied in automotive perception and motion-parameter estimation [1,2]. However, during off-road operation, tracked vehicles are subjected to multiple excitation sources, including road-surface irregularities, track–wheel interactions, and structural responses of the suspension and vehicle body, resulting in a broadband and complex platform-vibration environment [3]. Existing studies have shown that vehicle vibration and complex platform motion can cause spectral distortion of FMCW millimeter-wave radar echoes and degradation of sensing performance [1,4]. Since the robustness evaluation of automotive radar requires such vibration effects to be investigated under controllable and repeatable conditions, it is necessary to construct a laboratory vibration environment based on measured vehicle-vibration characteristics that can reproduce their effects on radar performance.

Radar vibration measurement and platform-motion compensation improve the estimation or correction of motion from received signals, while random-vibration control reproduces prescribed mechanical spectra and statistical characteristics. Section 2 reviews these research directions, emphasizing recent studies and their relationship to laboratory reproduction of radar degradation. Matching mechanical vibration descriptors alone does not guarantee agreement of the nonlinear radar response.

The reconstruction problem addressed here is how to allocate permissible spectral mismatch when laboratory vibration inputs can only approximately reproduce the measured environment. A conventional aggregate PSD error does not explicitly prioritize deviations according to the radar processing response. The specific contribution is to translate an established weak-perturbation response into reference-weighted spectral descriptors and use them to constrain vibration-frequency distribution errors jointly with PSD and higher-order statistics. For a fixed operator, exact PSD equality already implies REWS equality; the additional constraint changes the priorities of approximate matching and does not add independent spectral information or determine the complete nonlinear radar response.

To address these issues, this paper proposes a radar-performance-oriented spectral operator and a statistically equivalent vibration-reconstruction method. A small-amplitude single-tone model first supplies reference frequency weights for finite-CPI Doppler processing. These weights are combined with the measured vibration PSD and displacement–phase mapping to construct REWS, which is introduced alongside conventional vibration statistics as a reconstruction constraint. Numerical simulations and shaker tests then assess whether the reconstructed inputs reproduce selected radar-response characteristics; such agreement is not assumed from REWS matching alone.

The main contributions of this paper are as follows:

1. A finite-CPI weak-perturbation response is formulated as a reference frequency weighting for performance-oriented vibration reconstruction.
2. A radar-performance-oriented spectral operator is introduced to map the measured LOS vibration spectrum into REWS and sensitive-band energy descriptors, providing reconstruction priorities beyond conventional vibration metrics.
3. The proposed spectral descriptors are incorporated into a statistically equivalent vibration-reconstruction method together with PSD and higher-order statistical constraints, and the reconstructed inputs are evaluated through numerical radar processing and controlled shaker tests.

## 2. Related Work

### 2.1. Radar Vibration Measurement and Platform-Motion Compensation

Vibration-induced echo phase and micro-Doppler components have established theoretical foundations [5,6], as does FMCW displacement–phase mapping [2,7]. Finite-CPI sampling and chirp repetition determine the slow-time spectral response [8]. Recent vibration-measurement studies build on these mechanisms to improve displacement estimation. Ciattaglia et al. [9] evaluated vibration measurements using automotive millimeter-wave radars. Ma et al. [10] combined radar and accelerometer measurements, using accelerometer-aided phase unwrapping and displacement fusion. Li et al. [11] combined optimized beat-frequency estimation with maximum likelihood phase estimation to improve vibration-displacement extraction in noise. Su et al. [12] investigated dynamic demodulation to improve vibration measurement in the presence of phase jumps, phase noise, and interference. Their primary output is an estimated displacement history or vibration parameter, whereas the present work generates mechanical excitation histories for laboratory testing.

Platform-motion compensation addresses the related problem of removing motion-induced artifacts from radar data. Poole et al. [13] used synchronized IMU measurements for frame-level deconvolution of vibration effects. Poole and Arbabian [14] implemented an accelerometer-assisted deconvolution-kernel generator for real-time compensation, and their subsequent anchor-based method [4] inferred multimode vibration and nonharmonic transient motion from stationary environmental references. These methods use inertial or radar-reference information to correct received signals and report performance through quantities such as sideband suppression and SNR improvement. Our reconstruction method uses measured LOS acceleration to generate test inputs and evaluates errors in reproduced spectral broadening and spurious-peak positions. The objectives and evaluation metrics differ, so the studies provide methodological context rather than a basis for direct numerical ranking. The established phase-modulation mechanism and frequency selectivity are used as reference-weighting foundations here, rather than claimed as new findings.

### 2.2. Random Vibration Environment Reconstruction

Random-vibration reconstruction and control seek agreement with prescribed mechanical environments. Earlier work addressed kurtosis control through IFFT phase manipulation [15], multi-axis target definition [16], and multi-exciter non-Gaussian control [17]. Recent methods extend the controlled descriptors and temporal characteristics. Zheng et al. [18] separated power-spectrum and kurtosis control for multi-shaker non-Gaussian testing. Ren et al. [19,20] investigated nonstationary non-Gaussian control and time-varying RMS environments. Zheng et al. [21] jointly controlled kurtosis and crest factor, while Zheng et al. [22] developed multi-channel control of spectral densities, correlation coefficients, skewnesses, and kurtoses. These studies improve mechanical-environment fidelity through spectral, distributional, temporal, or correlation constraints.

Here, radar-response reference weighting augments PSD and higher-order statistical matching by prioritizing frequency-distribution errors under a specified radar configuration. Exact PSD equality would also imply REWS equality for a fixed operator; the constraint is useful when approximate reconstruction leaves residual spectral errors. Radar-response agreement is evaluated separately through simulation and shaker tests.

## 3. Spectral Error Mechanism and Error-Transfer Model of FMCW Radar Under Vibration

### 3.1. FMCW Slow-Time Model with Vibration Disturbance

The transmitted signal of a frequency-modulated continuous-wave (FMCW) radar can be expressed as

(1) $$s_{\mathrm{tx}}\left(t\right)=A_{\mathrm{tx}}\exp\left\{j\left(2\pi f_{c}t+\pi kt^{2}+\phi_{0}\right)\right\},$$

where $A_{\mathrm{tx}}$ is the amplitude of the transmitted signal, $f_{c}$ is the carrier center frequency, $k=B/T_{\mathrm{chirp}}$ is the frequency-modulation slope, *B* is the sweep bandwidth, $T_{\mathrm{chirp}}$ is the duration of a single chirp, and $\phi_{0}$ is the initial phase.

The received signal can be expressed as

(2) $$s_{\mathrm{rx}}\left(t\right)=A_{\mathrm{rx}}\exp\left\{j\left(2\pi f_{c}\left(t-\tau_{0}\right)+\pi k{\left(t-\tau_{0}\right)}^{2}+\phi_{0}\right)\right\}.$$

Here, $A_{\mathrm{rx}}$ is the received-signal amplitude and $\tau_{0}$ is the round-trip propagation delay of the target echo.

Conventional range and velocity estimation and range–Doppler processing for FMCW radar have been extensively studied [8,23]. Therefore, the basic transmission, reception, and mixing processes are not further elaborated here, and only the slow-time model relevant to platform-vibration error analysis is retained. Let the chirp repetition period be $T_{r}=T_{\mathrm{chirp}}+T_{\mathrm{idle}}$, and let each frame contain *M* chirps. The slow-time sampling instants and coherent processing interval (CPI) are then given by

(3) $$t_{m}=mT_{r},\;T_{\mathrm{CPI}}=MT_{r},\;m=0,1,\ldots ,M-1.$$

For the range cell containing the target, let its true Doppler frequency be $f_{D}$. With platform vibration taken into account, the slow-time complex envelope of the *m*-th chirp can be written as

(4) $$z\left[m\right]=\alpha \exp\left\{j\left(2\pi f_{D}mT_{r}+\phi_{v}\left[m\right]\right)\right\},$$

where α is the complex amplitude of the target and $\phi_{v}\left[m\right]$ is the additional phase disturbance introduced by platform vibration. Equation (4) shows that platform vibration directly affects Doppler processing by altering the inter-chirp phase evolution.

### 3.2. LOS-Equivalent Vibration and Echo Phase Modulation

**Figure 1:**
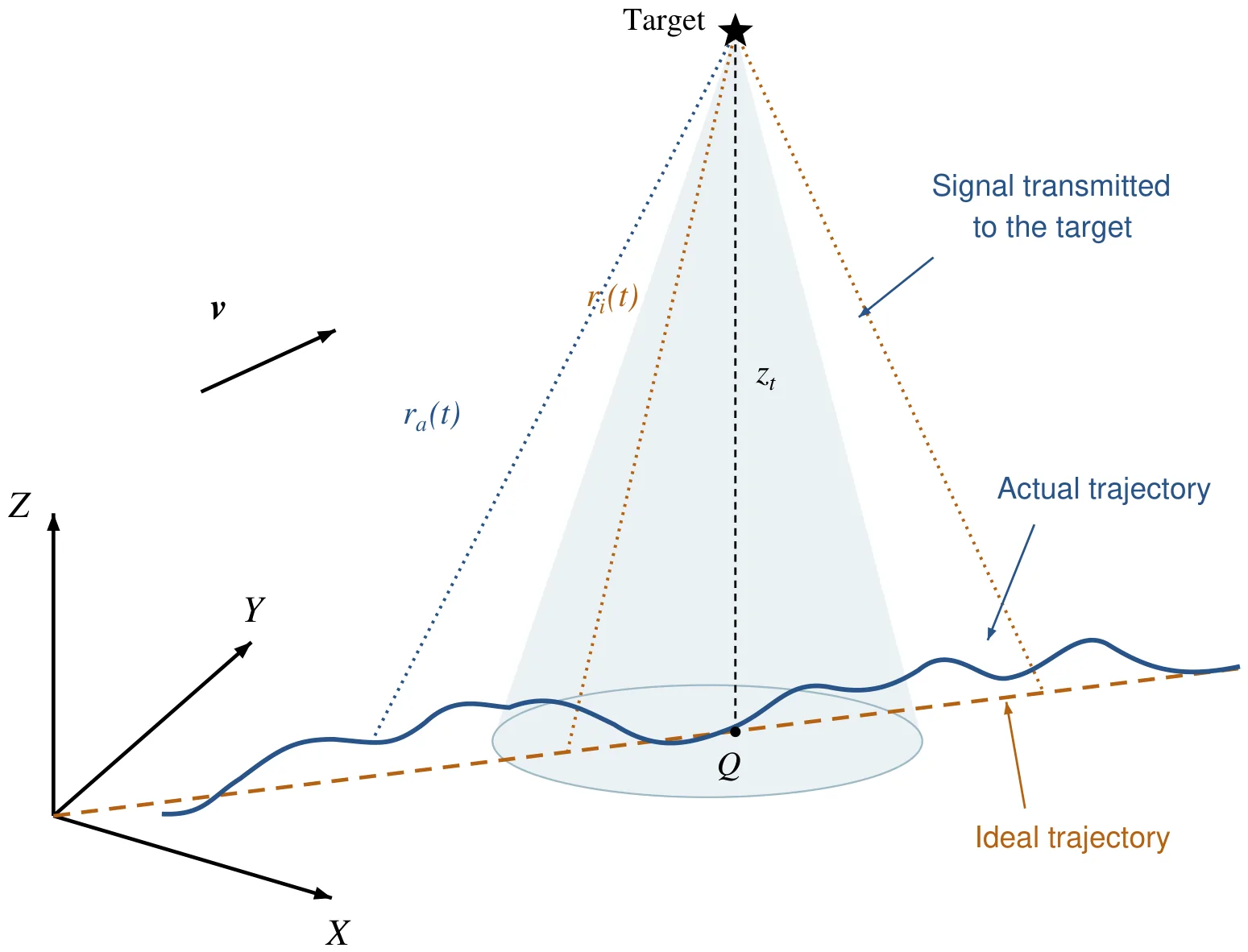
Schematic of the radar vibration condition and LOS-equivalent projection.

The effect of three-axis platform vibration on the radar echo depends on its projection along the target line of sight (LOS). As shown in Figure 1, let the vibration displacement at the radar mounting point be $d\left(t\right)={\left[d_{x}\left(t\right),d_{y}\left(t\right),d_{z}\left(t\right)\right]}^{T}$. Within a single CPI, when the target range is much larger than the vibration displacement and the variation in the LOS direction is small, the quasi-static approximation $u\left(t\right)\approx u_{0}$ can be adopted. The LOS-equivalent range disturbance induced by platform vibration is then expressed as

(5) $$\xi \left(t\right)\equiv \Delta R\left(t\right)\approx -u_{0}^{T}d\left(t\right).$$

For a monostatic radar, the variation in the round-trip propagation path is directly related to the LOS displacement, and a small displacement of the target or platform can be mapped to a change in the echo phase [2,7]. Therefore, the additional echo phase induced by platform vibration can be expressed as

(6) $$\phi_{v}\left(t\right)=\frac{4\pi }{\lambda }\xi \left(t\right),$$

where λ is the radar operating wavelength. The three-axis vibration with multiple frequency components is represented as

(7) $$d\left(t\right)=\sum_{i}A_{i}\sin\left(2\pi f_{i}t+\varphi_{i}\right),$$

where $A_{i}$ is the displacement-amplitude vector in meters, $f_{i}$ is the frequency in hertz, and $\varphi_{i}$ is the initial phase in radians of the *i*-th component. The corresponding phase disturbance can be expressed as

(8) $$\phi_{v}\left(t\right)=\sum_{i}\beta_{i}\sin\left(2\pi f_{i}t+\phi_{i}\right),\;\beta_{i}=\frac{4\pi }{\lambda }\left|u_{0}^{T}A_{i}\right|,$$

where $\beta_{i}$ is the dimensionless phase-modulation index of the *i*-th vibration component. The phase $\phi_{i}$ includes the sign of the LOS projection: $\phi_{i}=\varphi_{i}$ when $-u_{0}^{T}A_{i}>0$, and $\phi_{i}=\varphi_{i}+\pi$ when $-u_{0}^{T}A_{i}<0$, modulo 2π; a zero projection has zero modulation amplitude and an arbitrary phase. This relationship converts the three-axis mechanical vibration of the vehicle into an LOS phase disturbance acting on the FMCW slow-time signal, providing the input for the subsequent frequency-selectivity analysis.

### 3.3. Sensitive Frequency Bands and Frequency-Weighting Characteristics

To identify reference frequency bands for vibration reconstruction, this subsection considers a small-amplitude single-frequency LOS displacement. The purpose is to characterize finite-CPI frequency selectivity and construct a weak-perturbation reference weight, rather than to approximate the complete measured off-road response by a globally linear model. The displacement is written as

(9) $$\xi \left(t\right)=A\sin\left(2\pi f_{v}t+\varphi_{0}\right).$$

Here, *A* is the LOS displacement peak amplitude in meters, $f_{v}$ is the vibration frequency in hertz, and $\varphi_{0}$ is the initial phase in radians. According to Equation (6), the phase disturbance at the slow-time sampling instants is

(10) $$\phi_{v}\left[m\right]=\beta \sin\left(2\pi f_{v}mT_{r}+\varphi_{0}\right),\;\beta =\frac{4\pi A}{\lambda }.$$

The first-order approximation requires the overall LOS displacement excursion of the signal being linearized to be small on the phase scale: $\max_{m}\left|\phi_{v}\left[m\right]\right|\ll 1$, for which 4πA/λ≪1 is a sufficient condition in the single-tone case. Smallness relative to the target range alone is not sufficient. Under this condition,

(11) $$e^{j\phi_{v}\left[m\right]}\approx 1+j\beta \sin\left(2\pi f_{v}mT_{r}+\varphi_{0}\right).$$

The omitted complex-envelope remainder satisfies the pointwise bound

$$\left|e^{j\phi_{v}\left[m\right]}-1-j\phi_{v}\left[m\right]\right|\leq \frac{|\phi_{v}{\left[m\right]|}^{2}}{2}\leq \frac{\beta^{2}}{2}.$$

At 77GHz, λ≈3.90mm. For the processed Case D vehicle-vibration record, the isolated 100–500Hz component has a displacement RMS of 9.93µm and an absolute peak of 70.58 µm. These values correspond to a phase RMS of 0.0320rad and a peak modulation index of β≈0.228; the maximum first-order complex-envelope error is 0.0259. The measured high-frequency component therefore approximately satisfies the small-phase condition and supports use of the first-order model to construct the frequency-selective reference weights. For a sinusoidal acceleration of peak amplitude $a_{\mathrm{pk}}$, $A=a_{\mathrm{pk}}/{\left(2\pi f_{v}\right)}^{2}$, so the displacement associated with a fixed acceleration amplitude decreases quadratically with vibration frequency.

The exact Jacobi–Anger representation provides a complementary interpretation [24]:

$$e^{j\beta \sin\theta }=\sum_{\ell =-\infty }^{\infty }J_{\ell }\left(\beta \right)e^{j\ell \theta },\;\theta =2\pi f_{v}mT_{r}+\varphi_{0},$$

where $J_{\ell }$ is the Bessel function of the first kind. For small β, $J_{0}\left(\beta \right)=1-\beta^{2}/4+O\left(\beta^{4}\right)$, $J_{\pm 1}\left(\beta \right)=\pm \beta /2+O\left(\beta^{3}\right)$, and higher-order sideband amplitudes are $O(\beta^{\left|\ell \right|})$. Hence, Equation (11) retains the carrier and the leading sidebands to first order in amplitude. The identity $\sum_{\ell =-\infty }^{\infty }J_{\ell }^{2}\left(\beta \right)=1$ [25] gives the harmonic-coefficient energy outside orders 0,±1 as

$$1-J_{0}^{2}\left(\beta \right)-2J_{1}^{2}\left(\beta \right)=\frac{\beta^{4}}{32}+O\left(\beta^{6}\right).$$

This coefficient-energy measure characterizes the omitted higher-order sidebands in the weak-modulation regime. Together with the measured high-frequency phase scale above, it supports the first-order model used to construct the reference frequency weights. The subsequent numerical echo generation retains the complete exponential phase factor, so the response-level evaluation includes the sampled multifrequency modulation without applying the linearized expression to the full record. Periodic vibration produces micro-Doppler sidebands whose spacing depends on the vibration frequency, as established by Chen et al. [5]. Harris [26] describes how a finite observation window determines DFT-channel response, spectral leakage, and off-bin attenuation. Together, these established mechanisms motivate the reference weighting used here: vibration frequency sets the sideband locations, and finite-CPI windowing determines their projection onto the selected Doppler channels. The particular sensitive bands below are derived for the processing configuration adopted in this study.

For a rectangular slow-time window of length *M*, its normalized frequency response can be expressed as

(12) $$W_{M}\left(\Delta f\right)=\frac{1}{M}e^{j\pi \left(M-1\right)\Delta fT_{r}}\frac{\sin(\pi MT_{r}\Delta f)}{\sin(\pi T_{r}\Delta f)},$$

where Δf is the frequency offset in hertz between a signal component and the frequency at which the window response is evaluated. For a general non-negative slow-time window w[m], the corresponding normalized response is

$$W_{w}\left(\Delta f\right)=\frac{\sum_{m=0}^{M-1}w\left[m\right]e^{j2\pi \Delta fmT_{r}}}{\sum_{m=0}^{M-1}w\left[m\right]}.$$

The rectangular response above is recovered with w[m]=1. The numerical reference weights in this study use the Hann window, so $W_{w}$ is used in the definitions below. The frequency grid of the slow-time DFT is

(13) $$f_{n}=\frac{n}{T_{\mathrm{CPI}}},\;n=0,1,2,\ldots .$$

For the isolated positive-frequency first-order sideband, the projection onto a selected Doppler-offset channel is strongest when the vibration frequency approaches that channel’s center $f_{n}$. Off-bin components are distributed across neighboring channels according to the finite observation window. This channel-specific response motivates the sensitive-band construction; it does not imply that the total energy summed over all Doppler bins has the same frequency selectivity or that frequencies outside the selected bands have no radar effect.

To describe this frequency selectivity, the normalized response function of the *n*-th sensitive channel is defined as

(14) $$G_{n}\left(f\right)={\left|W_{w}\left(f-f_{n}\right)\right|}^{2},$$

where $G_{n}\left(f_{n}\right)=1$. For the positive-frequency first-order sideband, its isolated contribution to the *n*-th channel scales as

(15) $$e_{n}\left(f_{v},A\right)\propto \beta^{2}G_{n}\left(f_{v}\right).$$

Equation (15) describes a reference sideband-power weighting, not the full output power or a velocity-estimation error. The negative-frequency sideband has the corresponding response $|W_{w}\left(-f_{v}-f_{n}\right){|}^{2}$; a complete channel-power calculation must retain both sidebands and their interference for a specified initial phase. The reference response $G_{n}$ is used here to identify frequency-selective channels and construct an approximate reconstruction weight.

**Figure 2:**
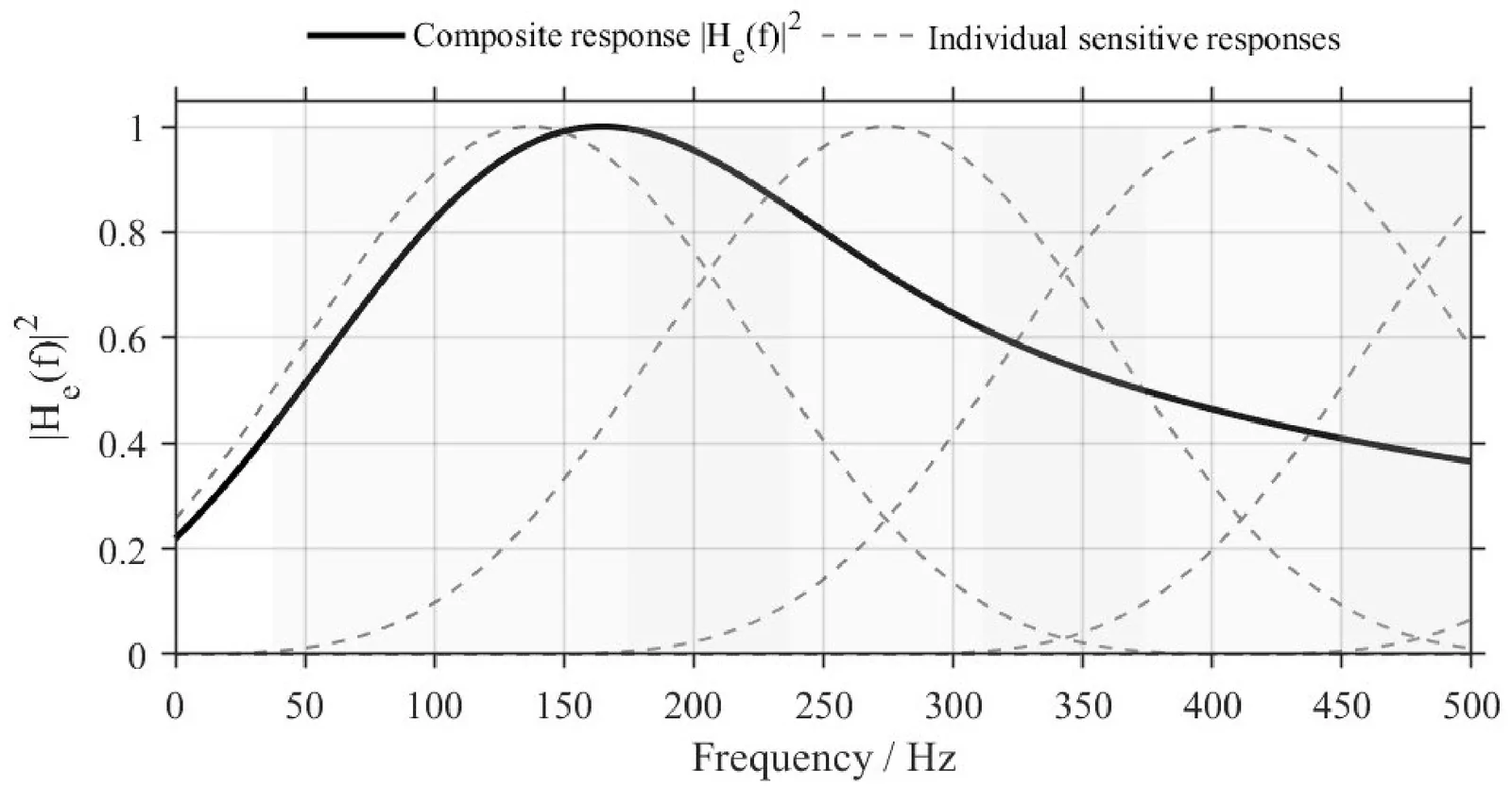
Performance-related equivalent frequency-weighting function and sensitive frequency bands.

Accordingly, the weak-perturbation reference frequency-weighting function used for reconstruction is denoted by $H_{e}\left(f\right)$. For a single reference channel,

(16) $$|H_{e}{\left(f\right)|}^{2}\propto G_{n}\left(f\right),$$

whereas, when multiple sensitive channels are considered, their responses can be combined using weights determined according to the velocity-domain performance metric of interest. For the radar parameters used in this study, $1/T_{\mathrm{CPI}}\approx 137\;\mathrm{Hz}$; therefore, the first nonzero sensitive center frequency is approximately $1/T_{\mathrm{CPI}}\approx 137\;\mathrm{Hz}$. Figure 2 illustrates the equivalent frequency-weighting function and the associated sensitive frequency bands.

The above single-tone analysis identifies the frequency selectivity of a weak-perturbation reference response. It does not establish independent or additive physical error contributions for strong broadband phase modulation. The next section combines this reference weighting with the measured vehicle-vibration PSD to prioritize spectral agreement during reconstruction; the resulting criterion is distinguished from the nonlinear radar response.

## 4. Radar-Performance-Oriented Spectral Operator Representation

The vibration environment of tracked vehicles under off-road operating conditions is essentially a broadband random process that deviates significantly from a Gaussian distribution. To theoretically quantify its influence on radar sensing systems, a single statistical measure or the original power spectral density (PSD) alone is insufficient to fully characterize its effects. In this section, the vibration environment is analyzed and parametrically described based on measured vehicle-test data, and a radar-performance-oriented spectral operator is constructed. Its output is defined as the Radar-Error-Weighted Spectrum (REWS), which provides target constraints for the subsequent statistically equivalent vibration reconstruction.

### 4.1. Vehicle Vibration Data Acquisition and Target Spectrum Construction

To construct a high-fidelity statistical-equivalence model, vehicle vibration data were first collected from a tracked vehicle operating on typical off-road terrain.

#### 4.1.1. Experimental Platform and Test-Condition Configuration

A specific type of tracked special-purpose vehicle was selected as the test platform. To cover the vibration environments that the radar system may encounter, combined test conditions involving different road-surface types and vehicle speeds were designed, as summarized in Table 1.

**Figure 3:**
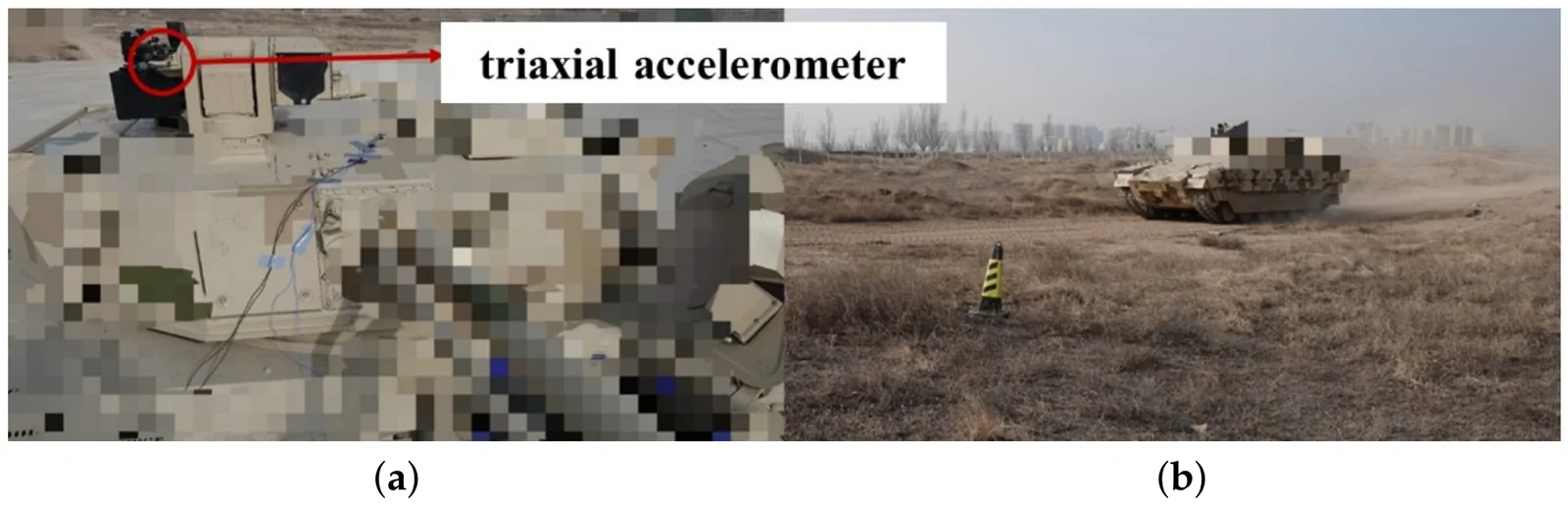
Vehicle off-road tests: (**a**) sensor installation position and (**b**) mixed off-road terrain test.

The vehicle tests are shown in Figure 3. A PCB 356A16 triaxial accelerometer (PCB Piezotronics, Inc., Depew, NY, USA), with a measurement range of ±50g and a frequency response of 0.5Hz–5kHz, was rigidly mounted on the sensing-system mounting base at the top of the vehicle body to accurately capture the vibration excitation experienced by the radar. An LMS SCM05 acquisition system (LMS International NV, Leuven, Belgium) was used with a sampling frequency of 4096Hz. The subsequent analysis in this study is mainly based on the data from Case D (mixed off-road terrain), which represents the most complex vibration environment among the tested conditions.

#### 4.1.2. Time-Domain Statistical Characterization

Analysis of the measured data shows that different driving conditions have a pronounced influence on the statistical distribution characteristics of the vibration signals. To quantify the vibration intensity, impulsiveness, and non-Gaussian characteristics under different operating conditions, the acceleration root mean square (RMS), kurtosis (*K*), skewness (*S*), and crest factor ($C_{F}$) were calculated from the three-axis time-domain vibration data for the four representative test conditions listed in Table 1. The results are summarized in Table 2.

As shown in Table 2, the vibration intensity and amplitude distributions in the three directions vary considerably among the different operating conditions. Case B exhibits relatively high kurtosis and crest-factor values, indicating strong transient impacts during vehicle maneuvering, whereas Case D is characterized by sustained broadband vibration under mixed off-road conditions. These results characterize the time-domain statistical properties of the measured vehicle-vibration environment and provide the statistical targets for the subsequent LOS-equivalent parameterization and vibration reconstruction.

### 4.2. Joint Parametric Representation Based on LOS Acceleration PSD and Time-Domain Statistical Features

Within the local range–phase model, the vibration input is represented by its linear component along a fixed reference LOS. Acceleration is the directly measured and controlled quantity in both vehicle testing and single-axis shaker reconstruction. The measured mounting-point record can include local motion generated by vehicle rotation; it is not a measurement of translation at the vehicle rotation center. The reconstruction nevertheless targets a scalar linear excitation along the reference LOS, rather than a reproduction of vehicle angular motion. Both the frequency-domain and time-domain statistical parameters are therefore defined in this reference LOS acceleration domain, whereas displacement and phase are intermediate quantities in the subsequent model-based mapping.

Under the fixed-coordinate and fixed-LOS approximation associated with Equation (5), the reference LOS acceleration is defined as

(17) $$a_{\mathrm{LOS}}\left(t\right)=u_{0}^{T}a\left(t\right),$$

where a(t) denotes the conditioned three-axis mounting-point vibration record and $u_{0}$ is the reference LOS unit vector expressed in the same coordinates. With $a\left(t\right)=\ddot{d}\left(t\right)$ under the fixed-LOS approximation, the sign convention in Equation (5) gives $\ddot{\xi }\left(t\right)=-a_{\mathrm{LOS}}\left(t\right)$; this sign does not affect the acceleration-to-displacement PSD relation. This fixed projection does not recover an attitude-corrected acceleration history. Differences between the measurement point and the radar phase center, and changes in the sensor-frame LOS, require additional installation-geometry and angular-motion information to quantify. If the cross-spectral density matrix of the measured three-axis acceleration is denoted by $S_{aa}\left(f\right)$, the PSD of the reference LOS acceleration is given by

(18) $$S_{a,\mathrm{LOS}}\left(f\right)≜S_{a_{\mathrm{LOS}}a_{\mathrm{LOS}}}\left(f\right)=u_{0}^{T}S_{aa}\left(f\right)u_{0}.$$

The acceleration PSDs are one-sided spectra for non-negative frequencies. Acceleration is expressed in $m\;s^{-2}$ in the spectral mapping, giving $S_{a,\mathrm{LOS}}$ units of ${\left(m\;s^{-2}\right)}^{2}/\mathrm{Hz}$. Values reported in g are converted using $1\;g=9.80665\;m\;s^{-2}$ before acceleration-to-displacement mapping; a PSD expressed in $g^{2}/\mathrm{Hz}$ is multiplied by the square of this conversion factor. Kurtosis, skewness, and crest factor are dimensionless.

The PSD primarily describes the second-order frequency-domain energy distribution of a random process. For vibration signals exhibiting pronounced transient and non-Gaussian characteristics, relying solely on the PSD is insufficient to fully preserve their time-domain statistical characteristics [27]. To simultaneously characterize the overall intensity, amplitude distribution, and impulsive characteristics of the LOS vibration, the RMS, kurtosis, skewness, and crest factor of $a_{\mathrm{LOS}}\left(t\right)$ are used for time-domain statistical characterization. The statistical feature vector is defined as

(19) $$m_{a}={\left[a_{\mathrm{RMS}},K,S,C_{F}\right]}^{T},$$

where $a_{\mathrm{RMS}}$, *K*, *S*, and $C_{F}$ denote the RMS, kurtosis, skewness, and crest factor of the LOS-equivalent acceleration, respectively. The same statistical notation is used throughout the reconstruction formulation; *S* without a frequency argument denotes skewness, whereas $S_{a}\left(f\right)$ denotes an acceleration PSD.

Accordingly, the basic joint parameter set describing the measured LOS vibration environment is defined as

(20) $$P_{\mathrm{LOS}}=\left\{S_{a,\mathrm{LOS}}\left(f\right),m_{a}\right\}.$$

This measurable joint set constrains mechanical spectral and amplitude-statistical fidelity, but does not distinguish the frequency-dependent effects of vibration on radar velocity estimates. This motivates the radar-oriented operator below.

### 4.3. Radar-Performance-Oriented Spectral Operator

Section 4.2 established the vibration-environment representation in the LOS acceleration domain. This subsection combines the acceleration-to-phase mapping with the weak-perturbation reference weighting from Section 3.3 to construct a performance-oriented spectral criterion for vibration reconstruction. It does not define an exact mapping from a broadband input PSD to nonlinear radar-performance degradation.

For nonzero frequencies, the acceleration-to-displacement relation gives $S_{\xi \xi }\left(f\right)=S_{a,\mathrm{LOS}}\left(f\right)/{\left(2\pi f\right)}^{4}$, followed by the displacement-to-phase mapping $\phi_{v}\left(t\right)=4\pi \xi \left(t\right)/\lambda$. This inverse relation is undefined at zero frequency: acceleration alone does not determine the constant displacement or constant-velocity component. Moreover, $H_{e}\left(0\right)$ need not vanish, so the reference response does not generally cancel the $1/f^{4}$ factor. The operator must therefore have an explicit domain bounded away from zero.

Let $\Omega_{n}$ be the connected half-power band containing the nonzero reference frequency $f_{n}$, as specified below. The radar-relevant analysis domain and its lower boundary are fixed as

$$F_{r}=\left(\overset{N_{b}}{\underset{n=1}{⋃}}\Omega_{n}\right)\cap \left(0,f_{H}\right],\;f_{L}=\inf F_{r}>0,$$

where $N_{b}$ is the number of sensitive frequency bands considered and $f_{H}$ is the upper frequency of the reconstruction domain. The condition $f_{L}>0$ is an explicit requirement of this definition and is checked for the chosen window and sensitivity threshold. On this domain, the band-limited acceleration-to-displacement inverse is

$$D_{F_{r}}\left(f\right)=\left\{\begin{array}{cc} {\left(j2\pi f\right)}^{-2}, & f\in F_{r},\\ 0, & f\notin F_{r}. \end{array}\right.$$

This inverse recovers only the displacement components in $F_{r}$; it does not estimate the complete low-frequency vehicle motion.

The phase-disturbance spectrum describes the input modulation intensity but does not encode the frequency selectivity of finite-CPI processing. The reference function $H_{e}\left(f\right)$ is therefore introduced as a fixed weight. Combining the band-limited inverse and displacement-to-phase mapping with this weighting, the radar-performance-oriented spectral operator is defined as

(21) $$S_{\mathrm{eff}}\left(f\right)=T_{e}\left\{S_{a,\mathrm{LOS}}\left(f\right)\right\}≜\left\{\begin{array}{cc} {\left(\frac{4\pi }{\lambda }\right)}^{2}\frac{S_{a,\mathrm{LOS}}\left(f\right)}{{\left(2\pi f\right)}^{4}}{\left|H_{e}\left(f\right)\right|}^{2}, & f\in F_{r},\\ 0, & f\notin F_{r}. \end{array}\right.$$

Here, $S_{\mathrm{eff}}\left(f\right)$ is referred to as the Radar-Error-Weighted Spectrum (REWS). It is a weighted input-spectrum indicator used to guide vibration reconstruction, with a physical reference supplied by the single-tone weak-perturbation analysis. Its frequency variable denotes the input vibration frequency. REWS is used as the reconstruction constraint, while the complete multifrequency radar response is evaluated separately from the full phase-modulated echo. With the dimensionless reference weight $H_{e}\left(f\right)$, REWS has units of ${\mathrm{rad}}^{2}/\mathrm{Hz}$ and its integrated band energies have units of ${\mathrm{rad}}^{2}$; these quantities are interpreted as weighted phase-spectrum descriptors.

The REWS operator is constructed for the vibration-environment reconstruction task. Under the specified laboratory bandwidth and spectral-matching tolerances, it assigns higher weights to vibration frequencies that couple strongly into the selected radar-processing channels and is used jointly with the measured acceleration PSD and time-domain statistics. The weighting follows the frequency dependence of vibration-induced sidebands and the finite-CPI channel response. Accordingly, REWS retains the single-tone reference response as a fixed and reproducible weighting for sensitive-band matching. The multifrequency intermodulation mechanism is treated in Section 5.2, and the complete exponential phase factor is retained in the numerical echo model for response-level comparison.

The band limitation provides a direct boundedness result. For $C_{\lambda }={\left(4\pi /\lambda \right)}^{2}$, bounded $H_{e}$, and an integrable input PSD on $F_{r}$,

$$\int_{F_{r}}S_{\mathrm{eff}}\left(f\right)\;df\leq \frac{C_{\lambda }{∥H_{e}∥}_{\infty }^{2}}{{\left(2\pi f_{L}\right)}^{4}}\int_{F_{r}}S_{a,\mathrm{LOS}}\left(f\right)\;df<\infty .$$

Similarly, for p=1,2, the fixed operator satisfies

$$∥T_{e}\left\{S_{a}\right\}{∥}_{L^{p}\left(F_{r}\right)}\;\leq \;\frac{C_{\lambda }{∥H_{e}∥}_{\infty }^{2}}{{\left(2\pi f_{L}\right)}^{4}}{∥S_{a}∥}_{L^{p}\left(F_{r}\right)}.$$

Thus, the band energies are finite for an $L^{1}$ PSD, and the squared-spectrum loss defined below is finite for $L^{2}$ PSDs with a nonzero reference denominator. No cancellation at f=0 is assumed.

Three frequency settings have different roles: the preprocessing high-pass frequency controls measured-signal conditioning; the displacement-integration lower limit $f_{\min}$ controls displacement reconstruction; and $f_{L}$ defines the REWS analysis domain. With the present Hann reference response and half-power threshold, the saved frequency grid gives $f_{L}\approx 37.5\;\mathrm{Hz}$; the reconstruction domain has $f_{H}=500\;\mathrm{Hz}$. These bands are fixed for the measured target and all reconstructed inputs. For any $0<f_{\min}<f_{L}$,

$$1_{F_{r}}\left(f\right)1_{\left[f_{\min},\infty \right)}\left(f\right)=1_{F_{r}}\left(f\right).$$

**Figure 4:**
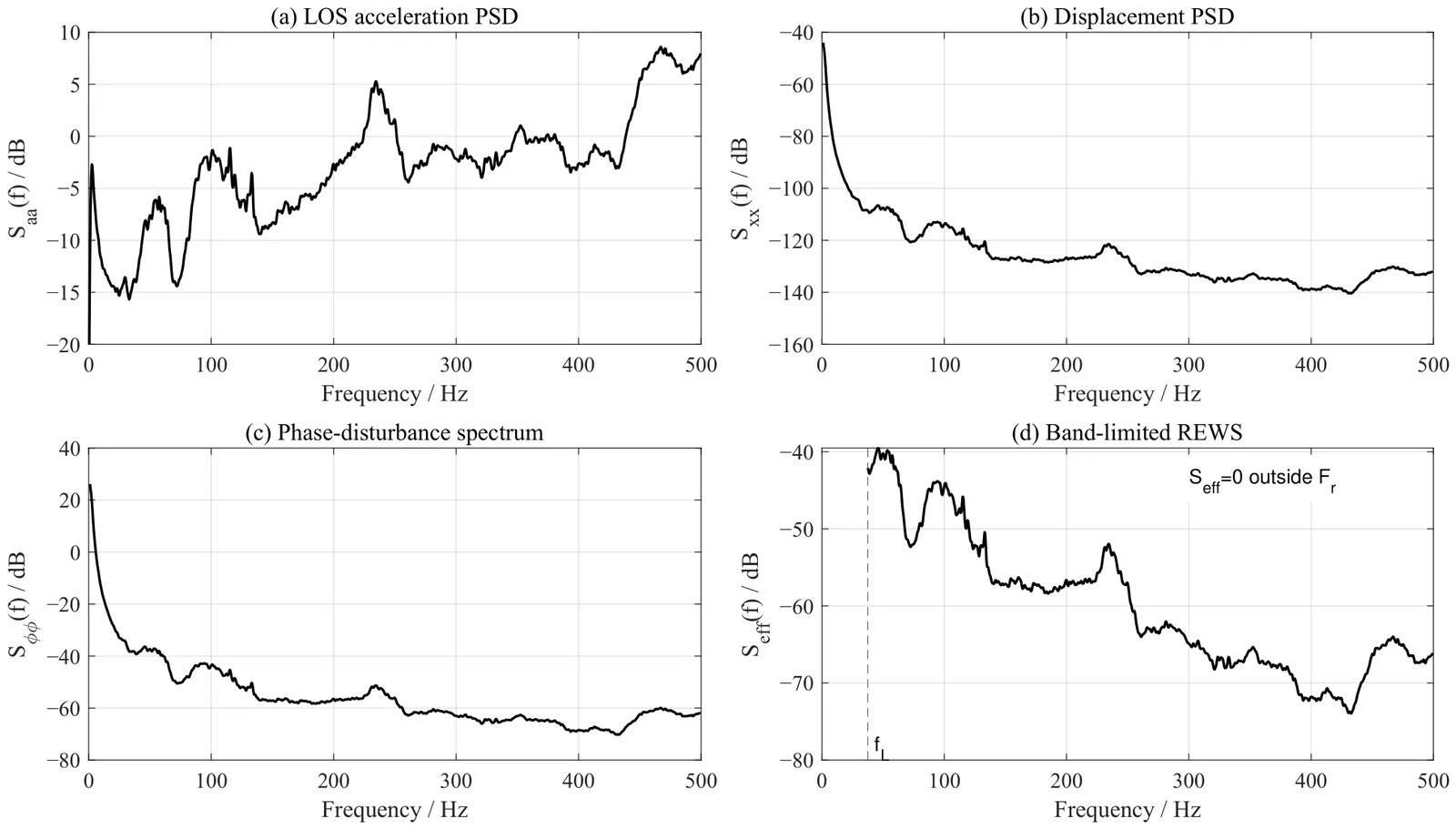
Spectral transfer relationship and band-limited REWS. Panels (**a**–**c**) retain the acceleration spectrum and the displacement–phase mapping with the existing integration cutoff. Panel (**d**) shows REWS only on the fixed sensitive domain $F_{r}$, with $f_{L}\approx 37.5\;\mathrm{Hz}$ and upper bound 500Hz. REWS is zero outside this domain; zero values are not drawn on the logarithmic axis.

Consequently, applying such a numerical integration cutoff to a fixed input PSD cannot change REWS, its band energies, or the REWS losses. This invariance does not apply when the measured signal is re-filtered, the sensitivity threshold or window is changed, or the cutoff enters $F_{r}$. Low-frequency motion outside $F_{r}$ remains part of the mechanical-vibration description and the complete response-level assessment; its radar effects are not represented by this band-limited indicator. Figure 4 illustrates the spectral transfer relationship and the resulting band-limited REWS.

Unlike an unweighted PSD-matching criterion, REWS assigns different reconstruction priorities to different input frequencies through the fixed displacement–phase and reference-response factors. A larger weighted contribution indicates greater importance within this reconstruction criterion; it does not establish a universal monotonic relationship with velocity bias, spectral broadening, or spurious-peak generation.

Based on the sensitive frequencies given in Section 3.3, the *n*-th sensitive center frequency is defined as

(22) $$f_{n}=\frac{n}{T_{\mathrm{CPI}}},\;n=1,2,\ldots .$$

The corresponding sensitive frequency band $\Omega_{n}$ is the connected component containing $f_{n}$ of the following individual-channel superlevel set:

(23) $$\Omega_{n}={\mathrm{cc}}_{f_{n}}\left\{f:G_{n}\left(f\right)\geq \gamma G_{n}\left(f_{n}\right)\right\}.$$

Here, ${\mathrm{cc}}_{f_{n}}$ selects the connected component containing $f_{n}$, thereby excluding remote sidelobes and periodic replicas. The dimensionless sensitivity threshold is denoted by γ, distinct from the target complex amplitude α. The numerical implementation uses γ=0.5, corresponding to the 3dB half-power boundary. Other thresholds change the analysis domain and require a renewed check that $f_{L}>0$. Each energy integral below is restricted to $\Omega_{n}\cap \left(0,f_{H}\right]$ when a band extends beyond the reconstruction range.

On this basis, the reference-weighted spectral energy within the *n*-th sensitive frequency band is defined as

(24) $$E_{n}^{\mathrm{eff}}=\int_{\Omega_{n}}S_{\mathrm{eff}}\left(f\right)\;df.$$

To compare the relative weighted contributions of the sensitive frequency bands within this criterion, the normalized effective-energy ratio is further defined as

(25) $$\rho_{n}^{\mathrm{eff}}=\frac{E_{n}^{\mathrm{eff}}}{\sum_{k=1}^{N_{b}}E_{k}^{\mathrm{eff}}}.$$

Here, $N_{b}$ denotes the number of sensitive frequency bands, distinct from the kurtosis *K*. A larger $\rho_{n}^{\mathrm{eff}}$ denotes a larger contribution to the REWS-based criterion, rather than a measured fraction of the actual radar error. If the bands overlap, these normalized entries are band descriptors and should not be interpreted as a disjoint partition of physical output energy.

Accordingly, $S_{a,\mathrm{LOS}}\left(f\right)$ and $m_{a}$ describe the measured mechanical vibration, while $S_{\mathrm{eff}}\left(f\right)$ and $E_{n}^{\mathrm{eff}}$ provide additional performance-oriented reconstruction descriptors. Their role is to prioritize agreement in frequency regions emphasized by the weak-perturbation reference weight, instead of treating all spectral deviations uniformly. Matching these descriptors is not, by itself, a sufficient condition for equivalence of the complete nonlinear radar response; performance agreement must be assessed separately.

## 5. Statistically Equivalent Reconstruction Based on the Radar-Performance-Oriented Spectral Operator

The measured LOS-equivalent vibration is reconstructed by jointly matching its PSD, time-domain statistics, and REWS descriptors. The multi-objective formulation uses reference weights to prioritize finite spectral mismatches. For a fixed operator, exact PSD equality already implies REWS equality, so the additional constraint changes matching priorities rather than adding independent spectral information. Neither PSD nor REWS matching alone guarantees agreement of the nonlinear radar response.

### 5.1. Optimization Formulation of the Reconstruction Objective

Let the measured LOS-equivalent acceleration time history be denoted by $a_{\mathrm{LOS}}^{0}\left(t\right)$ and the reconstructed LOS-equivalent acceleration time history by ${\tilde{a}}_{\mathrm{LOS}}\left(t\right)$. Their corresponding power spectral densities are denoted by $S_{a}^{0}\left(f\right)$ and ${\tilde{S}}_{a}\left(f\right)$, respectively. Consistent with the measured-data analysis in Section 4.1, their time-domain statistical feature vectors are defined as

(26) $$m^{0}={\left[a_{\mathrm{RMS}}^{0},K^{0},S^{0},C_{F}^{0}\right]}^{T},\;\tilde{m}={\left[{\tilde{a}}_{\mathrm{RMS}},\tilde{K},\tilde{S},{\tilde{C}}_{F}\right]}^{T},$$

where $a_{\mathrm{RMS}}$, *K*, *S*, and $C_{F}$ denote the acceleration RMS, kurtosis, skewness, and crest factor, respectively, and are used to characterize vibration intensity, non-Gaussianity, and impulsive characteristics.

According to Equation (21), the performance-oriented effective spectra corresponding to the measured and reconstructed vibrations are denoted by $S_{\mathrm{eff}}^{0}\left(f\right)$ and ${\tilde{S}}_{\mathrm{eff}}\left(f\right)$, respectively. According to Equation (24), the reference-weighted spectral energies within the *n*-th radar-sensitive frequency band are denoted by $E_{n}^{0}$ and ${\tilde{E}}_{n}$, respectively. The vectors composed of the energies in all $N_{b}$ sensitive frequency bands are denoted by $E_{\mathrm{eff}}^{0}$ and ${\tilde{E}}_{\mathrm{eff}}$.

Accordingly, statistically equivalent vibration reconstruction can be formulated as

(27) $${\tilde{a}}_{\mathrm{LOS}}^{*}\left(t\right)=\arg\min_{{\tilde{a}}_{\mathrm{LOS}}\left(t\right)\in A}J\left({\tilde{a}}_{\mathrm{LOS}}\left(t\right)\right),$$

where A denotes the set of realizable inputs satisfying the physical constraints of the shaker, including acceleration, velocity, displacement, and operating-frequency limits.

The objective function is defined as

(28) $$J=w_{T}J_{T}+w_{F}J_{F}+w_{R}J_{R},$$

where $J_{T}$ is the time-domain statistical-consistency term, $J_{F}$ is the frequency-domain spectral-structure-consistency term, and $J_{R}$ is the radar-performance-oriented spectral-consistency term. The corresponding weighting coefficients are $w_{T}$, $w_{F}$, and $w_{R}$.

The time-domain statistical-consistency term is defined as

(29) $$J_{T}=\frac{{∥D_{T}\left(\tilde{m}-m^{0}\right)∥}_{2}^{2}}{{∥D_{T}m^{0}∥}_{2}^{2}},$$

where $D_{T}$ is a normalization weighting matrix used to balance the influences of statistical quantities with different scales, including RMS, kurtosis, skewness, and crest factor. This term is used to maintain consistency between the reconstructed vibration and the measured vehicle data in terms of time-domain amplitude distribution, non-Gaussianity, and impulsiveness.

The frequency-domain spectral-structure-consistency term is defined as

(30) $$J_{F}=\frac{\int_{f_{a,L}}^{f_{a,H}}{\left|{\tilde{S}}_{a}\left(f\right)-S_{a}^{0}\left(f\right)\right|}^{2}\;df}{\int_{f_{a,L}}^{f_{a,H}}{\left|S_{a}^{0}\left(f\right)\right|}^{2}\;df}.$$

Here, $\left[f_{a,L},f_{a,H}\right]$ is the acceleration-PSD reconstruction range, distinct from the displacement-integration cutoff $f_{\min}$ and the REWS domain $F_{r}$. This term is used to maintain consistency between the reconstructed vibration and the measured vehicle vibration in terms of the original LOS-equivalent acceleration PSD, thereby preserving the frequency-domain energy-distribution characteristics of the measured off-road vibration.

The radar-performance-oriented spectral-consistency term is defined as

(31) $$J_{R}=\eta J_{\mathrm{eff}}+\left(1-\eta \right)J_{\mathrm{band}},\;0\leq \eta \leq 1,$$

where $J_{\mathrm{eff}}$ constrains the overall consistency of the performance-oriented effective spectrum, whereas $J_{\mathrm{band}}$ constrains the reference-weighted energy within each sensitive band. Since the chosen reconstruction strategy emphasizes these band descriptors, η<0.5 is adopted to prioritize the matching accuracy of $J_{\mathrm{band}}$. This is a design choice within the reference-weighted criterion, rather than a consequence of an exact additive decomposition of radar-performance degradation.

The effective-spectrum-consistency term is defined as

(32) $$J_{\mathrm{eff}}=\frac{\int_{F_{r}}{\left|{\tilde{S}}_{\mathrm{eff}}\left(f\right)-S_{\mathrm{eff}}^{0}\left(f\right)\right|}^{2}\;df}{\int_{F_{r}}{\left|S_{\mathrm{eff}}^{0}\left(f\right)\right|}^{2}\;df},$$

where $F_{r}$ is the fixed union of sensitive bands clipped to the reconstruction range, as defined in Section 4.3. The reference denominator is assumed positive. Both target and reconstructed REWS are evaluated on this same domain, and no frequencies below $f_{L}$ contribute to this loss. For fixed input PSDs, the loss and the band-energy loss below are unchanged by any displacement-integration cutoff $0<f_{\min}<f_{L}$.

The sensitive-band-energy-consistency term is defined as

(33) $$J_{\mathrm{band}}=\frac{{∥D_{E}\left({\tilde{E}}_{\mathrm{eff}}-E_{\mathrm{eff}}^{0}\right)∥}_{2}^{2}}{{∥D_{E}E_{\mathrm{eff}}^{0}∥}_{2}^{2}},$$

where $D_{E}$ is the sensitive-band weighting matrix. According to the normalized effective-energy ratio defined in Equation (25), it can be expressed as

(34) $$D_{E}=\mathrm{diag}\left(\rho_{1}^{\mathrm{eff}},\rho_{2}^{\mathrm{eff}},\ldots ,\rho_{N_{b}}^{\mathrm{eff}}\right).$$

This term increases the weights of the frequency bands that make dominant contributions to radar-performance degradation, so that the reconstruction process preferentially preserves frequency components that contribute strongly to velocity-spectrum leakage, spurious-peak generation, and velocity-estimation ambiguity.

### 5.2. Generation Framework and Method for Statistically Equivalent Vibration

**Figure 5:**
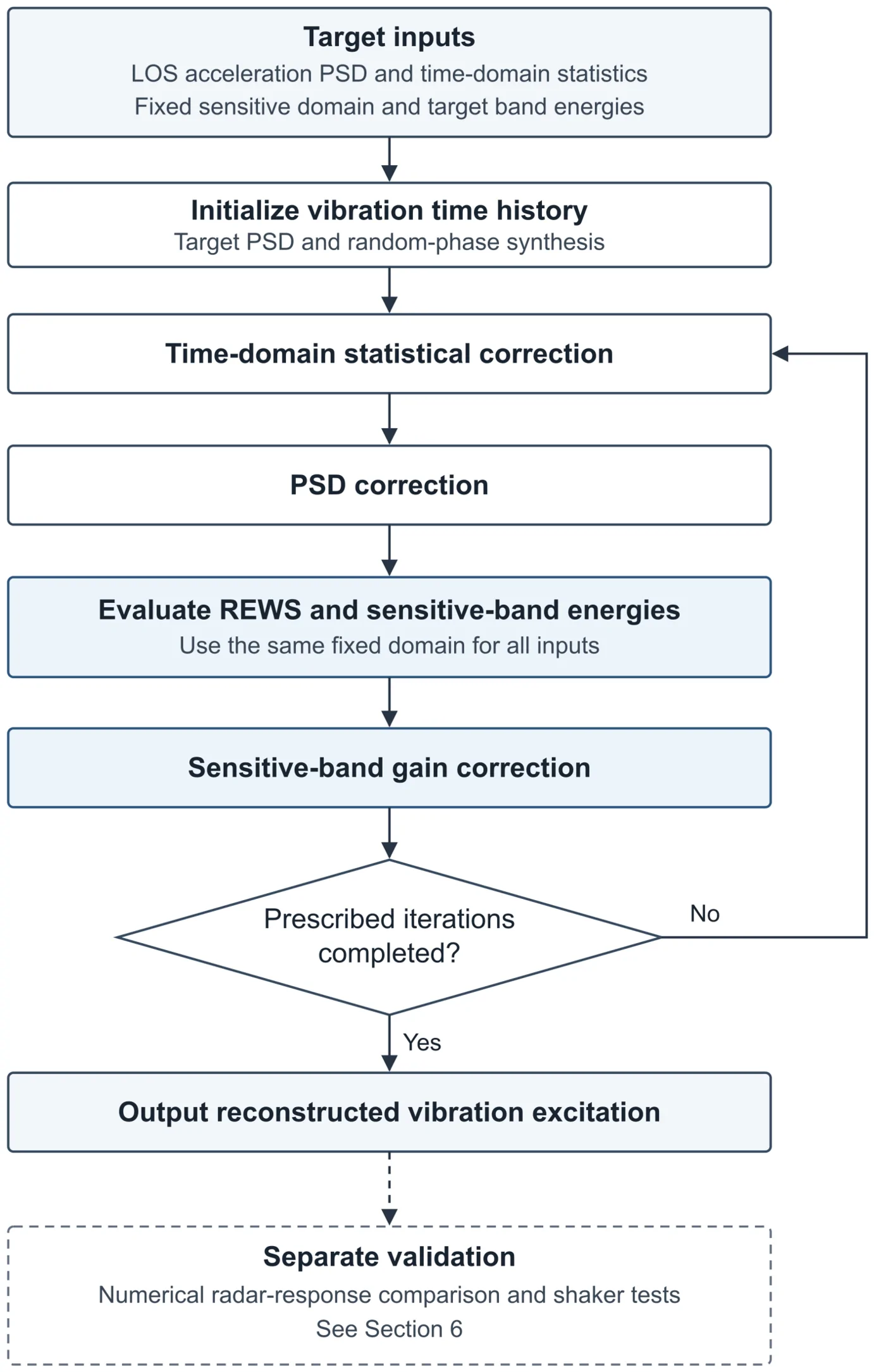
Flowchart of the equivalent-vibration generation algorithm.

Figure 5 summarizes the generation procedure associated with the objectives in Section 5.1: target-PSD synthesis, iterative statistical and PSD correction, and reference-weighted sensitive-band correction.

First, an initial random vibration time history is generated according to the target LOS-equivalent acceleration spectrum $S_{a}^{0}\left(f\right)$ obtained from the vehicle tests. Specifically, a frequency-domain synthesis method based on the target PSD and random phases is employed. At each discrete frequency point, the frequency-domain amplitude is determined according to the target spectrum, while random phases satisfying the conjugate-symmetry condition for a real-valued signal are assigned. The inverse fast Fourier transform (IFFT) is then applied to obtain the initial Gaussian random vibration sequence ${\tilde{a}}_{0}\left(t\right)$. Such target-spectrum-based random time-history generation methods have been widely used in random-vibration testing [15]. The resulting initial sequence approximately matches the target PSD in the frequency domain; however, its amplitude distribution is generally close to a Gaussian distribution and therefore cannot directly represent the non-Gaussian and impulsive characteristics of tracked-vehicle off-road vibration.

To make the time-domain statistical characteristics of the reconstructed vibration approach those of the measured vehicle data, a Hermite-polynomial-based nonlinear statistical correction is further applied to the initial sequence. Methods for constructing non-Gaussian random processes based on the Hermite moment models can be found in [28]. The corrected vibration sequence is expressed as

(35) $${\tilde{a}}_{1}\left(t\right)=\sum_{p=0}^{P_{H}}h_{p}H_{p}\left[{\tilde{a}}_{0}\left(t\right)\right],$$

where $P_{H}$ is the Hermite expansion truncation order, *p* is the polynomial-order index, $H_{p}\left(\cdot \right)$ is the *p*-th-order Hermite polynomial, and $h_{p}$ is the coefficient to be determined. The truncation order $P_{H}$ is distinct from the radar chirp count *M*. By adjusting $h_{p}$ together with the subsequent iterative correction, the RMS, kurtosis, skewness, and crest factor of the corrected sequence gradually approach the target statistical characteristics $m^{0}$ of the measured vehicle vibration. Because nonlinear statistical correction may cause local changes in the PSD, iteration between time-domain statistical correction and frequency-domain spectral correction is still required during the generation process to simultaneously satisfy the time-domain statistical constraints and frequency-domain spectral-structure constraints [18].

After a reconstructed sequence satisfying the basic time-domain statistical and original-PSD constraints is obtained, the radar-sensitive frequency bands are further corrected according to the performance-oriented spectral operator defined in Section 4.3. For the reconstructed spectrum ${\tilde{S}}_{a}^{\left(q\right)}\left(f\right)$ obtained at the *q*-th iteration, its performance-oriented effective spectrum is

(36) $${\tilde{S}}_{\mathrm{eff}}^{\left(q\right)}\left(f\right)=T_{e}\left\{{\tilde{S}}_{a}^{\left(q\right)}\left(f\right)\right\}.$$

The corresponding reference-weighted spectral energy within the *n*-th sensitive frequency band is

(37) $${\tilde{E}}_{n}^{\left(q\right)}=\int_{\Omega_{n}}{\tilde{S}}_{\mathrm{eff}}^{\left(q\right)}\left(f\right)\;df.$$

If ${\tilde{E}}_{n}^{\left(q\right)}$ deviates from the measured target value $E_{n}^{0}$, a band-gain factor is introduced within the corresponding sensitive frequency band for correction:

(38) $${\tilde{S}}_{a}^{\left(q+1\right)}\left(f\right)=g_{n}^{\left(q\right)}{\tilde{S}}_{a}^{\left(q\right)}\left(f\right),\;f\in \Omega_{n}.$$

Here, $g_{n}^{\left(q\right)}$ can be defined as

(39) $$g_{n}^{\left(q\right)}={\left(\frac{E_{n}^{0}}{{\tilde{E}}_{n}^{\left(q\right)}}\right)}^{\mu },\;0<\mu \leq 1,$$

where μ is a relaxation factor used to prevent excessive band-energy correction from causing oscillations in the spectral shape. When μ=1, the correction is directly determined by the ratio of the target energy to the current energy. When μ<1, the correction process becomes smoother and is suitable for cases involving strong coupling among multiple frequency bands or limited dynamic response of the shaker.

The correction is intended to improve agreement with the measured input in both the original LOS acceleration PSD and the reference-weighted descriptors ${\tilde{S}}_{\mathrm{eff}}\left(f\right)$ and ${\tilde{E}}_{n}$. Whether this agreement also reproduces the radar response is assessed separately in Section 6.3; it is not inferred solely from the reconstruction objective.

Off-road vibration of tracked vehicles is generally generated by multiple excitation sources, including low-frequency vehicle-body attitude response, mid-frequency structural vibration, and high-frequency track-engagement impacts. For multifrequency random vibration, the LOS-equivalent acceleration spectrum can be represented as the superposition of several frequency-band components:

(40) $$S_{a}\left(f\right)=\sum_{k=1}^{K_{b}}S_{a,k}\left(f\right),$$

where $S_{a,k}\left(f\right)$ denotes the restriction of the same PSD to the *k*-th band of a nonoverlapping partition and $K_{b}$ is the number of such bands. This bookkeeping partition is distinct from the possibly overlapping sensitivity bands $\Omega_{n}$; it is not an assumption that physically distinct excitation sources are uncorrelated.

For a fixed $H_{e}\left(f\right)$ and fixed analysis domain $F_{r}$, Equation (21) is a pointwise linear weighting of the input PSD. Consequently, the following identity holds exactly for the defined indicator, independently of whether the actual echo response is weakly or strongly modulated:

(41) $$T_{e}\left\{S_{a}\left(f\right)\right\}=\sum_{k=1}^{K_{b}}T_{e}\left\{S_{a,k}\left(f\right)\right\}.$$

Therefore, the total reference-weighted spectral energy over $F_{r}$ can be expressed as

(42) $$E_{\mathrm{eff}}=\int_{F_{r}}T_{e}\left\{S_{a}\left(f\right)\right\}\;df=\sum_{k=1}^{K_{b}}\int_{F_{r}}T_{e}\left\{S_{a,k}\left(f\right)\right\}\;df.$$

Equation (42) expresses the additive band decomposition of the reconstruction indicator. The same reference weights are applied to the target and reconstructed inputs, providing a consistent measure of their agreement in the selected sensitive bands. The nonlinear radar response is evaluated separately with the complete phase-modulated echo model.

Nonlinear multifrequency response and role of the reconstruction indicator.

To distinguish this algebraic property from the physical response, consider *L* components in Equation (8), with initial phases $\theta_{r}$. The Jacobi–Anger expansion gives the exact slow-time representation

$$\begin{array}{cc} z[m] & =\alpha \sum_{k\in Z^{L}}C_{k}e^{j2\pi \left(f_{D}+k\cdot f\right)mT_{r}},\\ C_{k} & =\prod_{r=1}^{L}J_{k_{r}}\left(\beta_{r}\right)e^{j\sum_{r=1}^{L}k_{r}\theta_{r}},\;k\cdot f=\sum_{r=1}^{L}k_{r}f_{r}. \end{array}$$

Terms with at least two nonzero indices describe intermodulation between vibration components. For the rectangular window used in the reference derivation, the finite-CPI spectrum is

$$Z_{M}\left(\nu \right)=\alpha \sum_{k}C_{k}W_{M}\left(f_{D}+k\cdot f-\nu \right).$$

Its squared magnitude contains both individual combination-frequency contributions and cross terms between them. Coincident or unresolved components must therefore be combined coherently for a given record; another slow-time window replaces $W_{M}$ by its corresponding frequency response. The sampled spectrum is periodic with period $1/T_{r}$, so combination frequencies are also subject to slow-time aliasing.

The phase factor has unit magnitude, so these effects redistribute echo energy rather than create additional total energy. For two tones with independently uniform initial phases, the phase-averaged fraction associated with terms having both indices nonzero follows from the Bessel square-sum identity [25]:

$$\begin{array}{cc} \eta_{\mathrm{mix}}\; & =\sum_{k_{1}\neq 0}\sum_{k_{2}\neq 0}J_{k_{1}}^{2}\left(\beta_{1}\right)J_{k_{2}}^{2}\left(\beta_{2}\right)\\ \; & =\left[1-J_{0}^{2}\left(\beta_{1}\right)\right]\left[1-J_{0}^{2}\left(\beta_{2}\right)\right]\\ \; & =\frac{\beta_{1}^{2}\beta_{2}^{2}}{4}+O\left(\beta_{1}^{4}\beta_{2}^{2}+\beta_{1}^{2}\beta_{2}^{4}\right),\;\beta_{1},\beta_{2}\to 0. \end{array}$$

Thus, intermodulation is higher order in the weak-modulation limit and becomes increasingly relevant as the modulation depth grows. For the phase ensemble used in the derivation, phase averaging removes cross-interference terms while retaining combination-frequency power. In particular, when only $\beta_{2}$ is small, $\eta_{\mathrm{mix}}\approx \left[1-J_{0}^{2}\left(\beta_{1}\right)\right]\beta_{2}^{2}/2$, which quantifies its coupling to a stronger background component.

The added multifrequency analysis identifies the combination-frequency components and quantifies their coefficient energy. It complements the reconstruction objective: the exact expansion explains the intermodulation mechanism, while the single-tone analysis supplies fixed reference weights for agreement in selected sensitive bands, consistent with the frequency-dependent sideband mechanism [5] and finite-window channel response [26]. Numerical echo generation retains the complete exponential phase factor, so the response-level comparison includes the multifrequency coupling represented by the sampled model. This treatment gives the theoretical analysis, reconstruction objective, and response validation distinct and consistent roles.

After the generated statistically equivalent vibration signal is applied to the shaker, the actual table response may be affected by factors such as actuator bandwidth, fixture stiffness, closed-loop control accuracy, and load coupling. Therefore, the measured shaker-table response needs to be further verified. Let the actual shaker response be denoted by $a_{\mathrm{sh}}\left(t\right)$, with the corresponding LOS-equivalent PSD, time-domain statistical characteristics, and performance-oriented effective spectrum denoted by $S_{a,\mathrm{sh}}\left(f\right)$, $m_{\mathrm{sh}}$, and $S_{\mathrm{eff},\mathrm{sh}}\left(f\right)$, respectively. To avoid repeating the error formulations identical to those in Equations (30), (29), and (32), the three equivalence-evaluation metrics for the shaker response are written as

(43) $$\begin{array}{cc} \varepsilon_{F}\; & =J_{F}{|}_{{\tilde{S}}_{a}\to S_{a,\mathrm{sh}}},\\ \varepsilon_{T}\; & =J_{T}{|}_{\tilde{m}\to m_{\mathrm{sh}}},\\ \varepsilon_{R}\; & =J_{\mathrm{eff}}{|}_{{\tilde{S}}_{\mathrm{eff}}\to S_{\mathrm{eff},\mathrm{sh}}}. \end{array}$$

When $\varepsilon_{F}$, $\varepsilon_{T}$, and $\varepsilon_{R}$ satisfy the predefined tolerances, agreement is established for the selected mechanical statistics and REWS descriptors. Agreement of radar-performance metrics requires a separate response-level comparison and is not guaranteed by these three reconstruction tolerances.

In summary, the method combines target-PSD synthesis, non-Gaussian correction, and reference-weighted band correction to construct a controllable laboratory excitation. The resulting input is evaluated using both reconstruction descriptors and radar-response metrics, preserving the distinction between a performance-oriented reconstruction criterion and a guarantee of nonlinear performance equivalence.

## 6. Simulation and Experiments

The numerical simulations were performed using MATLAB (R2023b; The MathWorks, Inc., Natick, MA, USA). Numerical simulations compare the reconstructed vibration inputs and their radar responses with the original-input references. Shaker tests further assess laboratory implementation and measured-response consistency with the model-based radar reference.

### 6.1. Validation and Comparison Scheme

The comparison evaluates two levels: vibration-signal fidelity through PSD, time-domain statistics, and sensitive-band energy, and radar-response agreement through main-peak broadening and spurious-peak position. Table 3 lists the three reconstruction methods.

### 6.2. Vibration-Signal-Level Validation

**Figure 6:**
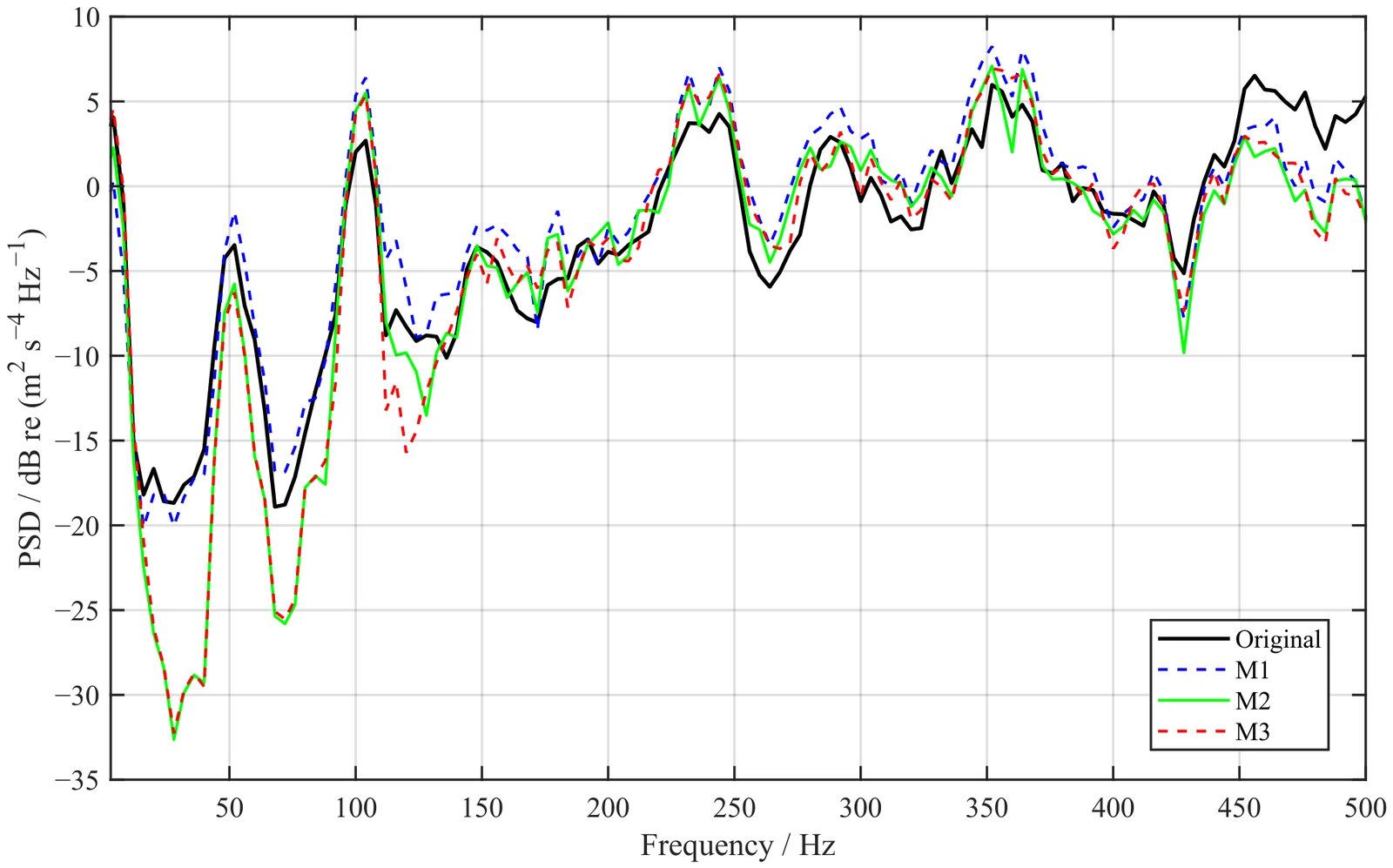
Comparison of PSDs between the original and reconstructed vibration signals.

Figure 6 compares the PSD of the original measured vehicle vibration with those reconstructed using the different methods. It can be seen that all three methods reproduce the overall shape of the target spectrum reasonably well.

The vibration-signal-level error metrics obtained using the different methods are listed in Table 4.

Figure 6 and Table 4 illustrate a trade-off between spectral and statistical matching. M1 closely reproduces the overall PSD and RMS, but its larger kurtosis, skewness, and crest-factor errors show that these second-order targets alone do not reproduce the selected non-Gaussian descriptors. Adding statistical constraints in M2 and M3 substantially reduces these errors, while their RMS errors increase to approximately 0.106 and 0.108, respectively. Thus, agreement in one descriptor should not be interpreted as uniform agreement in all vibration characteristics.

M3 is not superior to M2 in every statistical metric: its RMS and skewness errors are slightly higher, whereas its kurtosis and crest-factor errors are lower. The additional radar-oriented constraint is therefore evaluated as a way to redistribute the remaining spectral mismatch, rather than as a general improvement in all mechanical statistics. The subsequent sensitive-band and radar-response comparisons are needed to assess whether this trade-off benefits the intended radar test.

To quantify the reconstruction accuracy in the radar-sensitive frequency bands, the relative error of the sensitive-band effective-energy vector is defined as

(44) $$\varepsilon_{E}=\frac{{∥{\tilde{E}}_{\mathrm{eff}}-E_{\mathrm{eff}}^{0}∥}_{2}}{{∥E_{\mathrm{eff}}^{0}∥}_{2}},$$

where $E_{\mathrm{eff}}^{0}$ and ${\tilde{E}}_{\mathrm{eff}}$ denote the effective-energy vectors of the original and reconstructed vibration signals, respectively, with each element corresponding to one radar-sensitive frequency band. Five reconstruction realizations were generated using different random seeds, and the corresponding relative errors were averaged for comparison. The results are summarized in Table 5.

Unlike the weighted squared loss $J_{\mathrm{band}}$ used during reconstruction optimization, $\varepsilon_{E}$ is used here as an unweighted relative $L_{2}$ metric for comparing the reconstructed sensitive-band effective-energy distributions.

As shown in Table 5, the mean relative $L_{2}$ error decreases from 86.38% for M1 and 38.07% for M2 to 16.95% for M3. This result indicates that introducing the radar-performance-oriented spectral constraint improves the consistency of the effective-energy distribution across the radar-sensitive frequency bands.

The contrast with the similar overall PSD shapes in Figure 6 is important: the effective-energy vector weights local spectral differences by the specified radar response, so visually similar broadband spectra can yield different band-level errors. Under exact pointwise PSD matching, these weighted energies would also match; the benefit of the additional constraint arises when reconstruction leaves finite spectral errors. The reported mean covers five random-seed realizations, but without dispersion estimates, it does not establish statistical significance or a worst-case error bound.

### 6.3. Simulation Validation of Radar Performance

The simulation employs the FMCW radar spectral-error mechanism and error-transfer model under vibration established in Section 3, including echo generation, range FFT, slow-time Doppler FFT, and velocity-spectrum extraction. The vibration input is converted into an echo phase-modulation term through the LOS-equivalent displacement and is then applied to the slow-time signal. The numerical slow-time phase factor retains $e^{j\phi_{v}\left[m\right]}$ rather than replacing it by Equation (11); the first-order expansion is used only in the reference-weight construction. Retaining the exponential phase factor includes nonlinear phase modulation within this model, but does not prove that the REWS criterion completely represents it. The key simulation parameters are listed in Table 6.

**Figure 7:**
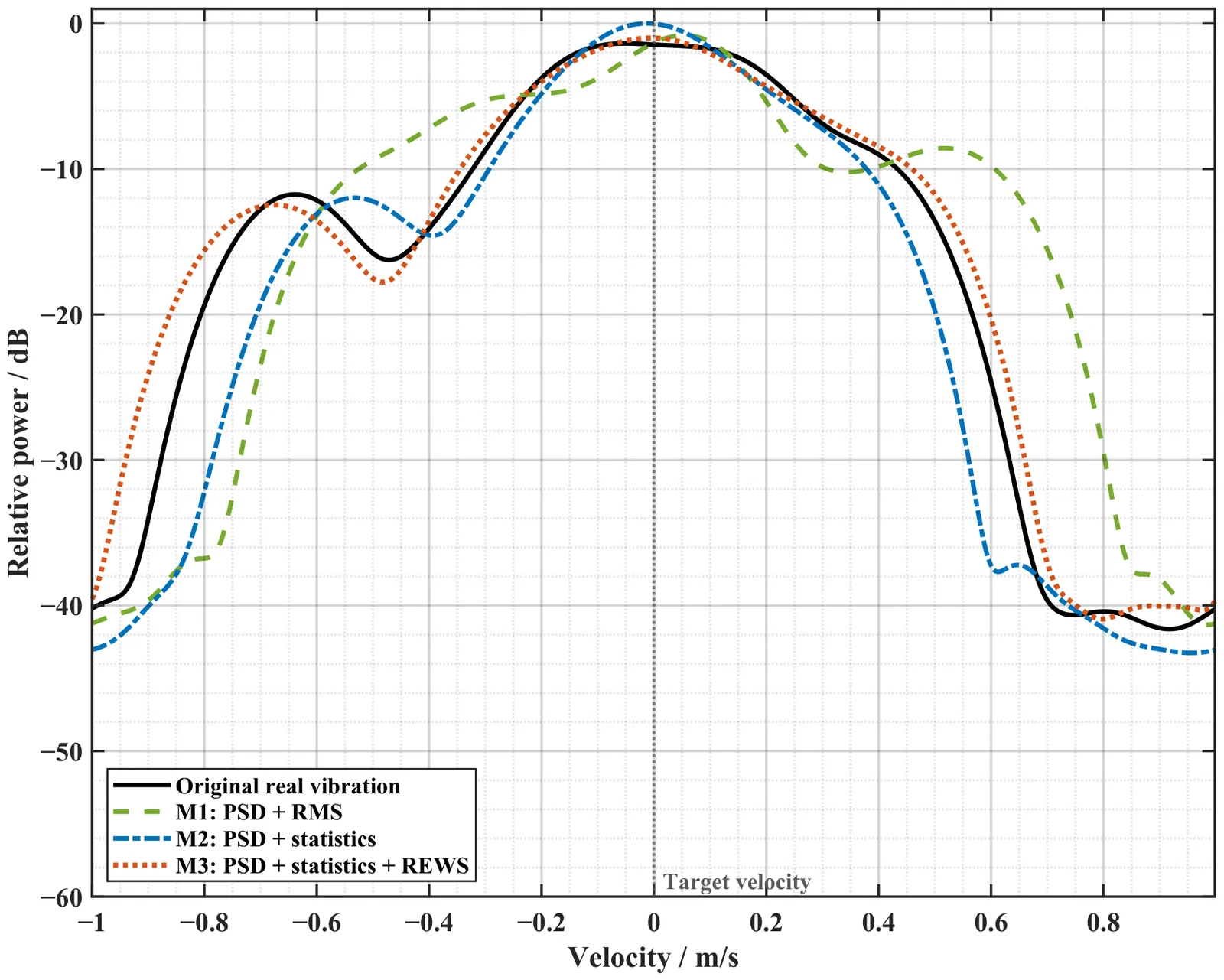
Comparison of velocity-spectrum slices under the original and reconstructed vibration inputs.

Figure 7 compares velocity-spectrum slices obtained using vibration inputs reconstructed by the different methods. The result obtained using the proposed M3 method is closer to that obtained using the original vibration input. For M2, noticeable deviations are observed in the spurious-peak position and main-peak broadening, although the overall trend of the main peak remains relatively close.

**Figure 8:**
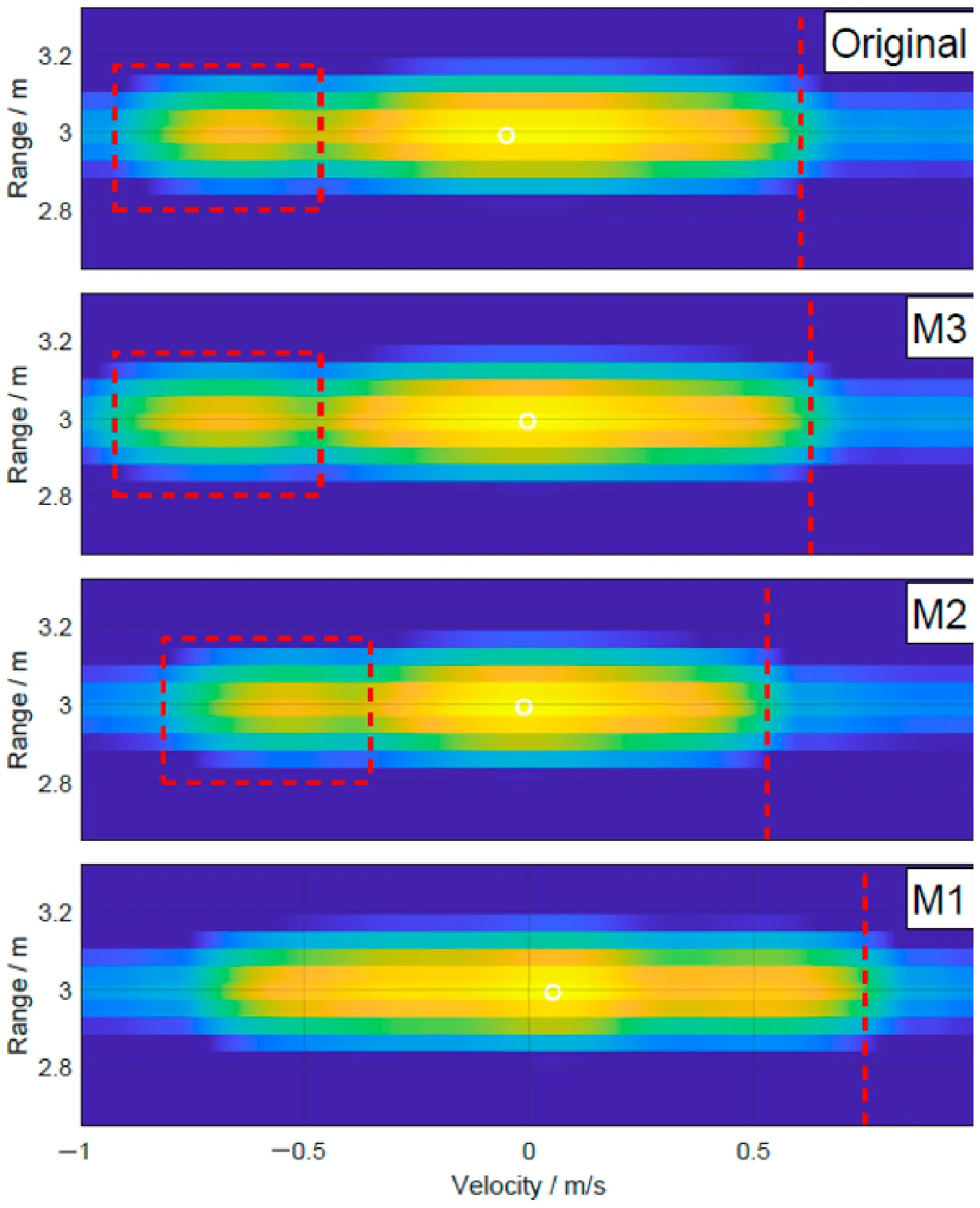
Comparison of range–Doppler maps under different vibration inputs. Red dashed boxes highlight spurious peaks, and red vertical dashed lines indicate main-peak broadening for comparison across the different methods.

Figure 8 compares the range–Doppler maps under different vibration inputs. The RD map corresponding to the proposed method is closer to that obtained using the measured vehicle-vibration input in terms of main-peak broadening and sidelobe distribution, indicating better equivalence in reproducing radar-performance degradation.

Table 7 separates two aspects of response reproduction: main-peak broadening describes the spread around the target response, whereas the spurious-peak center describes the location of a distinct vibration-induced component. Relative to the original-input simulation, M1 overestimates the broadening and does not produce an obvious spurious peak. The latter is not evidence of better reconstruction, because the reference response contains that feature; no peak-position error is assigned when a corresponding peak cannot be identified. M2 recovers an identifiable spurious component but underestimates both its center position and the main-peak broadening. M3 is closer to the reference in both metrics, with relative errors of 4.13% and 1.45%, compared with 12.40% and 15.94% for M2.

This ordering is consistent with the smaller sensitive-band energy error of M3. In this comparison, the more accurate energy distribution in the radar-sensitive bands produces a velocity spectrum closer to the original-vibration response and improves the reproduction of both main-peak broadening and spurious-peak position. This explains the benefit of the radar-oriented term when the overall PSD and selected statistical characteristics are already similar.

### 6.4. Shaker Tests and Comparison with a Model-Based Reference

**Figure 9:**
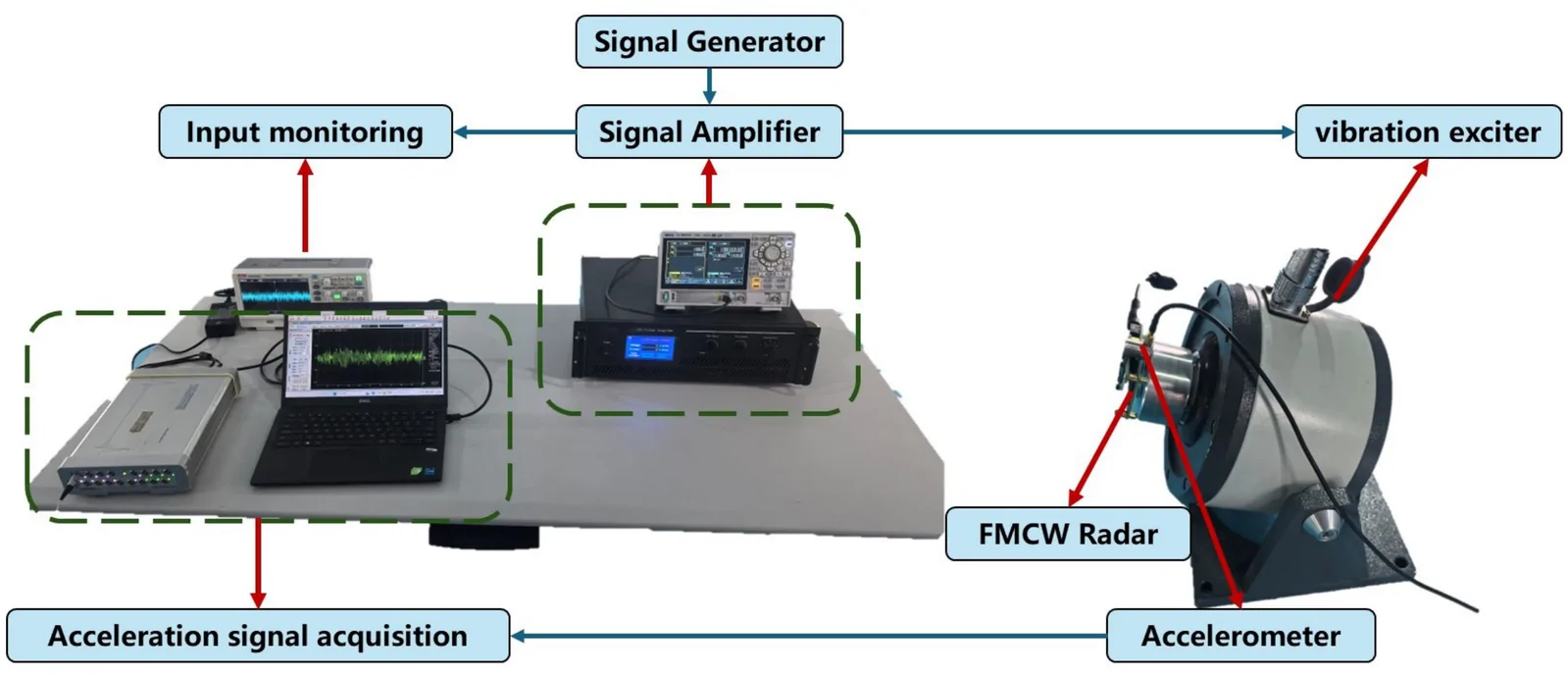
Schematic of the vibration-test platform and signal chain.

Because the available shaker cannot accurately replay the original broadband, impulsive vehicle-vibration record, reconstructed excitations are used to assess laboratory implementation and the measured radar response. Figure 9 shows the platform, comprising a signal generator, power amplifier, shaker, FMCW radar, acceleration sensor, and data-acquisition system. The setup used a VT-150 shaker and an LA-200 power amplifier (both from YMC Piezotronics Inc., Yangzhou, China), driven by a DG852 Pro signal generator (RIGOL Technologies Co., Ltd., Suzhou, China).

Input/response monitoring is used to verify the excitation. The radar parameters are consistent with Section 6.3, and a fixed corner reflector along the shaker vibration direction provides a stationary-target scenario. This single-axis test implements the reference LOS linear-vibration reconstruction, not vehicle pitch, roll, or a complete six-degree-of-freedom motion history.

The experimental IWR1843 ran the manufacturer’s demo software from mmWave SDK (version 3.4.0; Texas Instruments Incorporated, Dallas, TX, USA) without additional user calibration and used its built-in I/Q mismatch calibration [29,30] to mitigate a known source of phase distortion [31,32]. The host interface was mmWave Demo Visualizer (version 3.4.0; Texas Instruments Incorporated, Dallas, TX, USA).

**Figure 10:**
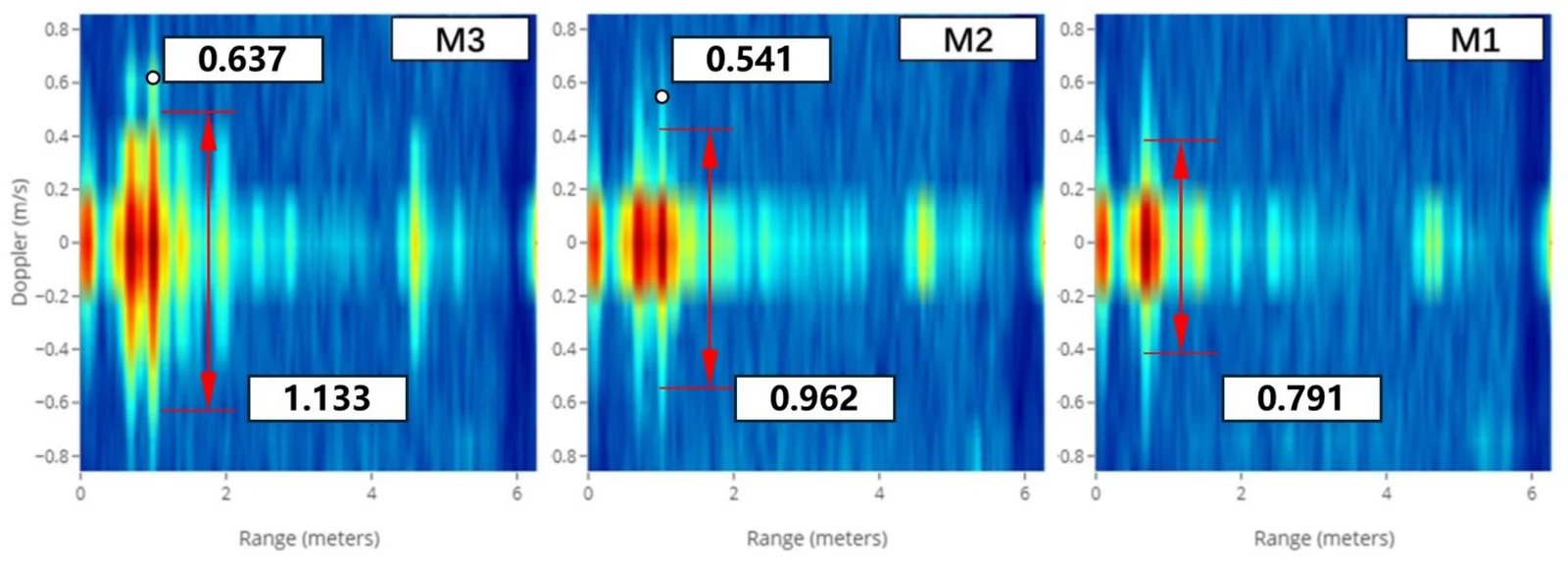
Radar results obtained in the shaker tests.

The excitations reconstructed by M1, M2, and M3 are separately applied to the shaker, and the resulting measured radar RD maps are compared under the controlled target configuration. The vehicle tests provided the measured vibration records, but no corresponding field radar measurements are available as a comparison baseline. Furthermore, direct accurate replay of the original record on the available shaker is not feasible, so a measured radar reference under original-record replay is also unavailable. The radar response simulated using the original measured vibration input is therefore used as a model-based reference. The resulting comparison assesses agreement of the measured hardware responses with that reference; it does not determine their accuracy relative to actual field radar degradation. The radar RD-map results obtained in the shaker tests are shown in Figure 10.

With reference to the simulation results obtained using the original measured vibration input in Table 7, the measured response under M3 has a main-peak broadening of 1.133m/s and a spurious-peak center position of 0.637m/s, corresponding to relative deviations of 6.36% and 7.68%, respectively, from the model-based reference. Compared with the other methods, these results show closer agreement with that reference for the evaluated conditions. These percentages are not reproduction errors relative to a field-measured radar response.

The measured M3 broadening and spurious-peak center are both below the model-based reference, whereas the simulated M3 broadening is above it and its peak center is closer to it. This difference illustrates why input-level matching and numerical response agreement cannot replace hardware testing. Shaker and mounting transfer characteristics, receiver imperfections, and model simplifications are possible contributors, but the present comparison does not separate their effects. The remaining deviations should therefore not be attributed solely to the reconstruction algorithm.

The measurements provide hardware-level evidence through the shaker, mounting structure, and radar receiver chain, but the choice of reference remains dependent on the radar model. Consequently, the present experiments cannot independently establish reproduction accuracy for actual field radar degradation. Synchronized field vibration and radar measurements, with an appropriate target-motion reference, are needed to assess that accuracy and distinguish vibration effects from other vehicle and scene effects.

## 7. Conclusions

This study introduced radar-response reference weighting into statistically equivalent vibration reconstruction, alongside PSD and higher-order statistical constraints. The method prioritizes frequency-distribution errors within practical reconstruction tolerances. In the evaluated cases, it maintains comparable agreement in the selected vibration statistics while reducing the mean sensitive-band effective-energy error from 38.07% for M2 to 16.95% for M3.

Improved sensitive-band energy matching also improves reproduction of radar-performance degradation in simulation. Relative errors in main-peak broadening and spurious-peak center position decrease from 12.40% and 15.94% for M2 to 4.13% and 1.45% for M3, respectively, against the original-vibration response. In shaker tests, the corresponding M3 measurements deviate from the model-based reference by 6.36% and 7.68%, demonstrating measured-response consistency under the evaluated conditions.

The reconstruction targets local linear vibration along a fixed reference LOS for a single-axis shaker. Mounting-point measurements can include rotation-induced local motion, but the method does not reproduce time-varying attitude, coordinate projection, or pointing-dependent scattering; phase-center equivalence also requires negligible differential motion in the analyzed band. The first-order model supplies reference weights, whereas strong multifrequency coupling is evaluated through the complete response model rather than REWS alone. Future work should test stronger modulation and attitude variation, and use synchronized field vibration and radar data with a target-motion reference to assess field degradation directly.

## Figures and Tables

**Table 1:** Vehicle vibration test conditions.

Case	Road Type	Test Description
Case A	Paved road	Stationary idling condition ($v<5\;\mathrm{km}\;h^{-1}$), used to obtain vehicle-body vibration data under engine excitation alone.
Case B	Paved road	Acceleration condition (v=0–$60\;\mathrm{km}\;h^{-1}$), covering vibration-excitation characteristics over different vehicle speeds.
Case C	Paved road	Constant-speed condition ($v=40\;\mathrm{km}\;h^{-1}$), used to obtain vehicle-body vibration data under high-speed operation.
Case D	Mixed off-road terrain	Constant-speed condition ($v=40\;\mathrm{km}\;h^{-1}$), exciting broadband random vibration and high-frequency track-engagement impacts.

**Table 2:** Comparison of statistical characteristics of three-axis vibration signals under different test conditions.

Case	Direction	RMS (g)	Kurtosis (*K*)	Skewness (*S*)	Crest Factor ($C_{F}$)
Case A Idling	X	0.07	2.89	−0.01	4.56
	Y	0.16	2.91	0.11	4.63
	Z	0.07	2.94	−0.07	4.77
Case B Acceleration	X	2.96	7.22	−0.12	17.42
	Y	4.89	5.70	−0.03	10.02
	Z	3.52	5.68	−0.01	13.62
Case C Paved-road cruising	X	2.40	4.14	0.03	6.02
	Y	4.46	3.91	−0.04	5.63
	Z	3.37	3.85	0.02	6.29
Case D Mixed off-road	X	2.78	4.74	0.09	7.21
	Y	4.86	3.80	−0.07	4.12
	Z	3.66	3.98	−0.01	5.47

**Table 3:** Comparison of different vibration-reconstruction methods.

Method	Reconstruction Targets	Key Feature
M1	PSD and RMS	Conventional spectral matching
M2	PSD, RMS, kurtosis, skewness, and CF	Non-Gaussian statistical matching
M3	PSD, RMS, kurtosis, skewness, CF, and REWS	Radar-performance-oriented matching

**Table 4:** Comparison of vibration-signal-level metrics for different reconstruction methods.

Method	RMS Error	Kurtosis Error	Skewness Error	CF Error
M1	$1.82\times 10^{-16}$	0.1711	0.9771	0.1484
M2	0.1059	0.0103	0.0499	0.1114
M3	0.1079	0.0059	0.0684	0.0330

**Table 5:** Comparison of sensitive-band effective-energy reconstruction errors.

Method	Mean Relative $L_{2}$ Error (%)
M1	86.38
M2	38.07
M3	16.95

**Table 6:** Key FMCW radar simulation parameters.

Parameter	Symbol	Value
Carrier frequency	$f_{c}$	77GHz
Sweep bandwidth	*B*	2GHz
Chirp duration	$T_{\mathrm{chirp}}$	50 µs
Idle time	$T_{\mathrm{idle}}$	7 µs
Number of chirps per frame	*M*	128
First sensitive frequency	$1/T_{\mathrm{CPI}}$	137Hz

**Table 7:** Comparison of range–Doppler spectral characteristics for different reconstruction methods.

Method	Main-Peak Broadening/(m/s)	Spurious-Peak Center Position/(m/s)
Original	1.21	0.69
M1	1.52	No obvious spurious peak
M2	1.06	0.58
M3	1.26	0.68

## Data Availability

The simulation and experimental data presented in this study are available from the corresponding author upon reasonable request.

## Abbreviations

The following abbreviations are used in this manuscript:

FMCW	Frequency-modulated continuous-wave
PSD	Power spectral density
RMS	Root mean square
LOS	Line of sight
CPI	Coherent processing interval
DFT	Discrete Fourier transform
FFT	Fast Fourier transform
IFFT	Inverse fast Fourier transform
REWS	Radar-Error-Weighted Spectrum
CF	Crest factor
RD	Range–Doppler

## References

[1] Hau F., Baumgärtner F., Vossiek M. (2020). The degradation of automotive radar sensor signals caused by vehicle vibrations and other nonlinear movements. Sensors.

[2] Xiong Y., Peng Z., Xing G., Zhang W., Meng G. (2018). Accurate and robust displacement measurement for FMCW radar vibration monitoring. IEEE Sens. J..

[3] Wang P., Rui X., Liu F., Wang G., Yu H., He B., Gu J. (2023). Generation mechanism and control of high-frequency vibration for tracked vehicles. Int. J. Mech. Syst. Dyn..

[4] Poole N., Arbabian A. (2024). Anchor-based, real-time motion compensation for high-resolution mmWave radar. IEEE J. Microwaves.

[5] Chen V.C., Li F., Ho S.S., Wechsler H. (2006). Micro-Doppler effect in radar: Phenomenon, model, and simulation study. IEEE Trans. Aerosp. Electron. Syst..

[6] Rüegg M., Meier E., Nüesch D. (2007). Vibration and rotation in millimeter-wave SAR. IEEE Trans. Geosci. Remote Sens..

[7] Anghel A., Vasile G., Cacoveanu R., Ioana C., Ciochina S. (2014). Short-range wideband FMCW radar for millimetric displacement measurements. IEEE Trans. Geosci. Remote Sens..

[8] Neemat S., Uysal F., Krasnov O., Yarovoy A. (2019). Reconfigurable range-Doppler processing and range resolution improvement for FMCW radar. IEEE Sens. J..

[9] Ciattaglia G., De Santis A., Disha D., Spinsante S., Castellini P., Gambi E. (2021). Performance evaluation of vibrational measurements through mmWave automotive radars. Remote Sens..

[10] Ma Z., Choi J., Yang L., Sohn H. (2023). Structural displacement estimation using accelerometer and FMCW millimeter wave radar. Mech. Syst. Signal Process..

[11] Li M., Zhang K., Xu H. (2025). Robust vibration displacement extraction for FMCW radar by incorporating optimized beat frequency and maximum likelihood estimation. IEEE Sens. J..

[12] Su L., Yang Z., Wu S., Guan Y., Tian Y., Qiao B., Chen X. (2026). FMCW radar dynamic demodulation: Enabling accurate vibration measurement. Mech. Syst. Signal Process..

[13] Poole N., Hor S., Arbabian A. A real-time, frame-level platform vibration compensation approach for mmWave radar systems. Proceedings of the 2021 18th European Radar Conference (EuRAD).

[14] Poole N., Arbabian A. (2023). A 130-nm fusion-based deconvolution kernel generator IC for real-time mmWave radar motion compensation. IEEE Access.

[15] Steinwolf A. (2012). Random vibration testing with kurtosis control by IFFT phase manipulation. Mech. Syst. Signal Process..

[16] Musella U., D’Elia G., Carrella A., Peeters B., Mucchi E., Marulo F., Guillaume P. (2018). A minimum drives automatic target definition procedure for multi-axis random control testing. Mech. Syst. Signal Process..

[17] Zheng R., Chen H., Vandepitte D., Luo Z. (2019). Multi-exciter stationary non-Gaussian random vibration test with time domain randomization. Mech. Syst. Signal Process..

[18] Zheng R., Chen G., Chen H. (2022). Power spectrum and kurtosis separation method for multi-shaker non-Gaussian random vibration control. Mech. Syst. Signal Process..

[19] Ren S., Chen H., Zheng R. (2024). Modified time domain randomization technique for multi-shaker non-stationary non-Gaussian random vibration control. Mech. Syst. Signal Process..

[20] Ren S., Chen H., Zheng R. (2024). Multi-shaker time-varying root mean square non-stationary random vibration environmental control test. J. Sound Vib..

[21] Zheng R., Chen F., Wang G., Yang F. (2025). Kurtosis and crest factor simultaneous control for non-Gaussian random vibration test. Mech. Syst. Signal Process..

[22] Zheng R., Wang G., Yang F. (2025). Non-Gaussian random vibration test by control of multiple correlation coefficients, skewnesses, and kurtoses. Int. J. Mech. Syst. Dyn..

[23] Patole S.M., Torlak M., Wang D., Ali M. (2017). Automotive radars: A review of signal processing techniques. IEEE Signal Process. Mag..

[24] National Institute of Standards and Technology Digital Library of Mathematical Functions.

[25] National Institute of Standards and Technology Digital Library of Mathematical Functions.

[26] Harris F.J. (1978). On the use of windows for harmonic analysis with the discrete Fourier transform. Proc. IEEE.

[27] Antoni J. (2006). The spectral kurtosis: A useful tool for characterising non-stationary signals. Mech. Syst. Signal Process..

[28] Winterstein S.R. (1988). Nonlinear vibration models for extremes and fatigue. J. Eng. Mech..

[29] Texas Instruments (2024). IWR1843 Single-Chip 76 to 81GHz FMCW mmWave Sensor.

[30] Texas Instruments (2023). Self-Calibration in TI’s mmWave Radar Devices.

[31] Rodriguez D., Li C. A digital I/Q correction technique for a 125-GHz interferometric radar with sub-micrometer sensitivity. Proceedings of the 2019 IEEE MTT-S International Microwave Symposium (IMS).

[32] Cardillo E. (2026). Setup-independent quadrature imbalance calibration for microwave and millimeter-wave Doppler radars. IEEE Trans. Microw. Theory Tech..

